# Evolution of Hierarchically Porous Nickel Alumina Catalysts Studied by X‐Ray Ptychography

**DOI:** 10.1002/advs.202105432

**Published:** 2022-01-20

**Authors:** Sebastian Weber, Ana Diaz, Mirko Holler, Andreas Schropp, Mikhail Lyubomirskiy, Ken L. Abel, Maik Kahnt, Arno Jeromin, Satishkumar Kulkarni, Thomas F. Keller, Roger Gläser, Thomas L. Sheppard

**Affiliations:** ^1^ Institute for Chemical Technology and Polymer Chemistry Karlsruhe Institute of Technology (KIT) Engesserstr. 20 Karlsruhe 76131 Germany; ^2^ Institute of Catalysis Research and Technology Karlsruhe Institute of Technology (KIT) Hermann‐von‐Helmholtz‐Platz 1 Eggenstein‐Leopoldshafen 76344 Germany; ^3^ Paul Scherrer Institut Villigen PSI 5232 Switzerland; ^4^ Deutsches Elektronen‐Synchrotron DESY Notkestrasse 85 Hamburg 22607 Germany; ^5^ Institute of Chemical Technology Universität Leipzig Linnéstraße 3 Leipzig 04103 Germany; ^6^ MAX IV Laboratory Fotongatan 2 Lund 225 94 Sweden; ^7^ Centre for X‐ray and Nano Science (CXNS) Deutsches Elektronen‐Synchrotron DESY Notkestrasse 85 Hamburg 22607 Germany; ^8^ Physics Department University of Hamburg Hamburg 20355 Germany

**Keywords:** heterogeneous catalysis, hierarchical porosity, imaging, pore characterization, sol–gel method, X‐ray ptychography, X‐ray tomography

## Abstract

The synthesis of hierarchically porous materials usually requires complex experimental procedures, often based around extensive trial and error approaches. One common synthesis strategy is the sol–gel method, although the relation between synthesis parameters, material structure and function has not been widely explored. Here, in situ 2D hard X‐ray ptychography (XRP) and 3D ptychographic X‐ray computed tomography (PXCT) are applied to monitor the development of hierarchical porosity in Ni/Al_2_O_3_ and Al_2_O_3_ catalysts with connected meso‐ and macropore networks. In situ XRP allows to follow textural changes of a dried gel Ni/Al_2_O_3_ sample as a function of temperature during calcination, activation and CO_2_ methanation reaction. Complementary PXCT studies on dried gel particles of Ni/Al_2_O_3_ and Al_2_O_3_ provide quantitative information on pore structure, size distribution, and shape with 3D spatial resolution approaching 50 nm, while identical particles are imaged ex situ before and after calcination. The X‐ray imaging results are correlated with N_2_‐sorption, Hg porosimetry and He pycnometry pore characterization. Hard X‐ray nanotomography is highlighted to derive fine structural details including tortuosity, branching nodes, and closed pores, which are relevant in understanding transport phenomena during chemical reactions. XRP and PXCT are enabling technologies to understand complex synthesis pathways of porous materials.

## Graphical abstract

Hard X‐ray ptychography in in situ 2D and ex situ 3D modes is shown as an emerging technology to study textural and structural changes of porous materials during thermal treatment. The porosity changes from the dried gel to calcined state of hierarchical porous (Ni/)Al_2_O_3_ catalysts are investigated with sub‐100 nm resolution.

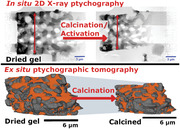

## 1 Introduction

Hierarchical structures are omnipresent in our environment and are particularly found in connection with transport processes, for example, the evolved human respiratory system for oxygen delivery in the lungs, or modern designed road networks for managing traffic flow.^[^ ^1^, ^2^ ^]^ Similarly to the above examples, mass transport properties strongly influence the performance of porous materials used in heterogeneous catalysis, particularly in terms of catalytic activity, product selectivity, and material stability. Diffusion limitations of reactants or products are highly relevant both in normal operating conditions and following changes induced by catalyst deactivation. Therefore, strategies have to be found for a knowledge‐based design of porous catalysts with optimized mass transport properties and improved catalyst performance. One promising approach is to design catalysts with hierarchical rather than uniform pore structures,^[^ ^3^, ^4^, ^5^, ^6^, ^7^ ^]^ generally including some combination of solids with micro‐ (<2 nm), meso‐ (2−50 nm), or macroporous (>50 nm) pore size distribution.^[^ ^5^, ^6^, ^7^, ^8^ ^]^ Here, we focus on meso‐/macroporous systems with the predominant transport processes of Knudsen diffusion in mesopores and molecular diffusion or convective flow in macropores.^[^ ^4^ ^]^ Introducing macropores into a uniform mesoporous system can provide higher mass transfer velocity by promoting convective flow over diffusion.^[^ ^3^, ^4^, ^9^ ^]^

The last two decades have brought significant progress in synthesis and design of meso‐/macroporous materials with potential applications in heterogeneous catalysis or adsorption.^[^ ^5^, ^7^, ^10^, ^11^, ^12^, ^13^, ^14^, ^15^, ^16^, ^17^, ^18^, ^19^ ^]^ Common synthesis strategies can be broadly divided into templating, post processing (often leaching), or self‐assembly techniques, each allowing for control over porosity, particle shape and being suited for certain material classes.^[^ ^5^, ^19^ ^]^ Modified sol–gel synthesis is a self‐assembly strategy to obtain powders or shaped particles with meso‐/macroporous structure in the form of spheres, membranes, or monoliths.^[^ ^15^, ^17^, ^19^, ^20^ ^]^ The sol–gel synthesis can be divided into five steps, each of which can influence the final texture and structure of the material:^[^ ^17^, ^20^, ^21^ ^]^ i) sol formation by hydrolysis and partial condensation, ii) gel formation via polycondensation, iii) aging of the gel, iv) drying of the gel, and v) stabilization, specifically calcination. A full understanding of sol–gel synthesis is challenging due to the many steps and parameters involved at each stage. Aside from introducing structure‐directing or phase separation agents (i.e., polymers), the final porosity can also be strongly determined by the solvent and its removal during drying or calcination.^[^ ^17^, ^20^ ^]^ The final calcination step can induce numerous changes to porosity, pore sizes, crystallization, sintering, partial, or even complete structural collapse. Obtaining greater understanding and control over these effects is highly desirable in the context of rational material design.^[^ ^7^, ^17^, ^20^ ^]^

Following synthesis, a further challenge is the accurate characterization of hierarchical porosity. Schlumberger et al. and Cychosz et al. reviewed gas sorption and Hg porosimetry as routine methods for analyzing hierarchical pore structures.^[^ ^22^, ^23^ ^]^ For meso‐/macroporous catalysts a combination of these techniques is necessary due to the different length scales probed, with Hg porosimetry normally used to measure macro‐ and mesopores down to 4 nm, and Ar‐ or N_2_‐sorption applied for smaller mesopores.^[^ ^22^, ^23^, ^24^, ^25^ ^]^ However, both methods have limited use in studying hierarchical pore structures, especially to retrieve more complex pore network information like connectivity or shape of individual pores or pore systems. Furthermore, gas sorption studies typically require thermal outgassing during sample preparation, which limits their application in studying dried gel samples and excludes their use in studying late stages of synthesis and some post‐synthesis treatment steps.^[^ ^22^, ^23^, ^24^ ^]^ In addition, Hg porosimetry is considered an invasive method, as probably not all Hg can be removed after the experiment and especially for fragile materials the sample may be damaged by high pressure intrusion of Hg.^[^ ^22^, ^23^, ^25^ ^]^ The application of conventional methods to study pore structure evolution especially during calcination is strictly limited, while in situ experiments are hardly feasible.

Alternatively, advanced imaging techniques based on X‐ray and electron tomography offer excellent opportunities to study textural and structural properties of hierarchically porous catalysts in a 3D spatially‐resolved manner.^[^ ^26^, ^27^, ^28^, ^29^ ^]^ As with conventional methods, meso‐/macroporous catalysts require a combination of imaging techniques to address all relevant length scales (nm–cm). Examples include combining electron tomography for nanoscale analysis, with focused ion beam‐scanning electron microscopy or X‐ray tomography for larger length scales.^[^ ^26^, ^30^, ^31^, ^32^, ^33^, ^34^, ^35^, ^36^ ^]^ Among reported X‐ray imaging techniques, hard X‐ray ptychography (XRP) and its 3D counterpart ptychographic X‐ray computed tomography (PXCT) are particularly promising. XRP and PXCT are relatively modern scanning coherent diffraction imaging techniques delivering high spatial resolutions (e.g., routinely <50 nm) limited neither by focusing optics (beam size) or by the scanning step size, and applicable to extended samples volumes (e.g., up to ≈100 µm diameter).^[^ ^37^, ^38^, ^39^, ^40^ ^]^ XRP and PXCT are capable of quantifying the local electron density (*N*_e_) of the sample in 2D and 3D space, respectively. This in turn provides sensitivity to mass density, which may in principle be used to retrieve chemical information.^[^ ^39^, ^40^, ^41^, ^42^ ^]^ The application of PXCT in catalysis research is still at a relatively early stage, although the potential of the method is clearly shown in several studies of textural/pore structures^[^ ^32^, ^33^, ^34^, ^35^, ^36^, ^43^, ^44^, ^45^, ^46^ ^]^ or chemical heterogeneity.^[^ ^32^, ^44^, ^45^, ^46^, ^47^, ^48^, ^49^, ^50^ ^]^ Recently, PXCT studies on PTFE membranes could derive porosity and tortuosity information, which was then used to calculate diffusion coefficients and permeability.^[^ ^51^ ^]^

**Figure 1:**
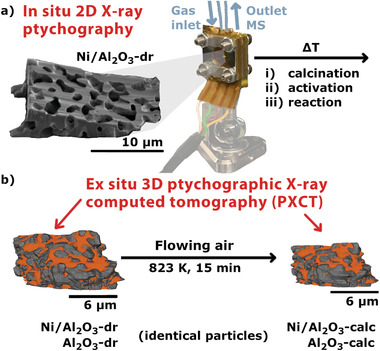
Overview of experiments performed. a) In situ 2D X‐ray ptychography of the dried gel Ni/Al_2_O_3_‐dr sample during calcination, activation, and reaction conditions in a nanoreactor setup. b) Illustration of the sample particles, which are placed on Cu‐pins for ex situ 3D ptychographic tomography experiments. Tomography data was acquired first on the dried gels (Ni/Al_2_O_3_‐dr and Al_2_O_3_‐dr), and again on the identical samples following calcination in a muffle furnace (Ni/Al_2_O_3_‐calc and Al_2_O_3_‐calc).

In this study, we present a combination of in situ 2D XRP and ex situ 3D PXCT to study the pore structure evolution of meso‐/macroporous Ni/Al_2_O_3_ catalysts during calcination (**Figure** **1**). The hierarchically porous Ni/Al_2_O_3_ catalyst has applications in dry reforming of CH_4_ and methanation of CO_2_,^[^ ^36^, ^52^ ^]^ showing improved conversion in the former process (89.5%) over a purely mesoporous catalyst (86.5%).^[^ ^52^ ^]^ Theoretical calculations of the effectiveness factor also suggest a superior performance of the meso‐/macroporous catalyst for methanation of CO_2_.^[^ ^36^ ^]^ In both cases, improved performance can be attributed to the hierarchical pore system, stimulating the present aim to investigate how hierarchical porosity can be accurately measured and quantified in complex solids. In situ XRP studies were performed in a nanoreactor setup,^[^ ^53^ ^]^ ensuring defined gas and temperature conditions during calcination of a dried gel Ni/Al_2_O_3_ sample. Complementary PXCT studies of identical dried gel particles of Ni/Al_2_O_3_ and Al_2_O_3_ before and after calcination were used to quantify the pore structure in 3D space. The X‐ray imaging results were compared with N_2_‐sorption, Hg porosimetry, and He pycnometry for bulk measurement of meso‐/macroporosity and solid skeletal density. A robust strategy is therefore presented based on XRP to study textural and structural changes at nanoscale resolution, and PXCT to quantitatively probe hierarchical porous features, in both cases producing data largely unavailable to contemporary methods. The methodology shown is broadly relevant for synthesis and characterization of materials with tailored pore structures.

## 2 Results and Discussion

### 2.1 Synthesis and Characterization of Hierarchically Porous Nickel Alumina Catalysts

Preparation of the hierarchically porous Ni/Al_2_O_3_ and Al_2_O_3_ catalysts was adapted from previous studies, reporting bimodal meso‐ and macroporous structures by sol–gel synthesis with polymerization induced phase separation.^[^ ^36^, ^52^, ^54^ ^]^ Briefly, chloride salts of Al and Ni were homogeneously mixed with poly(ethylene oxide) (PEO) in an ethanolic solution. Polymerization and phase separation were induced by addition of propylene oxide (PO) to obtain the wet gels, which were then dried and subsequently calcined.^[^ ^36^, ^52^, ^54^ ^]^ The dried gel samples discussed in this study (Ni/Al_2_O_3_‐dr and Al_2_O_3_‐dr) were separated from the main synthesis batch after drying, and therefore represent the catalyst state without subsequent treatment steps such as calcination, activation, or catalytic testing (see Supporting Information).

**Figure 2:**
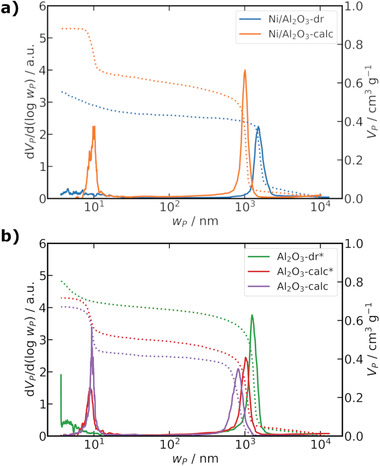
Hg porosimetry data for the a) Ni/Al_2_O_3_ and b) Al_2_O_3_ samples as dried gel and calcined. Al_2_O_3_‐dr* and Al_2_O_3_‐calc* are from a different sample batch than used for imaging studies. The solid lines show the pore width distribution as (d*V*_P_/d(log*w*_P_)) and the dashed lines the cumulative specific pore volume (*V*_P_).

Elemental analysis (C,H,N, Table S2, Supporting Information) revealed the presence of organic compounds and water in the dried gels, this was assigned to polymer or solvent residues from the synthesis and was not found in the calcined samples. Hg porosimetry studies (**Figure** **2**; Figure S1 and Table S1, Supporting Information) of the dried gel and calcined samples showed that only macropores (≈1 µm) were significantly present in the dried gels, while additional mesopores (≈10 nm) were conclusively identified only in the calcined samples.

The mode of the macropore width (*w*_P, M, Hg_) decreased after calcination from 1.5 to 1.0 µm in Ni/Al_2_O_3_, and from 1.2 to 1.0 µm in Al_2_O${}_{3}^{*}$. It should be noted that Hg porosimetry studies to compare the dried gel and calcined state of Al_2_O_3_ were carried out on a different sample batch than for the X‐ray imaging studies (indicated by an asterisk “*”). This was done due to an insufficient amount of sample remaining from the X‐ray imaging batch to allow both Hg porosimetry and N_2_ sorption, rather only Hg‐porosimetry on the X‐ray imaging batch was performed (Al_2_O_3_‐calc). To compensate for this, a new batch of dried gel sample (Al_2_O_3_‐dr*) was made by the same exact preparation procedure and examined by Hg porosimetry before and after calcination (Al_2_O_3_‐calc*). This might influence the absolute pore width values, as *w*_P, M, Hg_ for the same Al_2_O_3_‐calc batch as the X‐ray imaging studies was 0.82 µm, compared to 1.0 µm for the new batch (Al_2_O_3_‐calc*). Furthermore, small mesopore contributions (<10 nm) were found for Al_2_O_3_‐dr at *w*_P, M, Hg_ close to the resolution limit of Hg porosimetry. The Brunauer‐Emmett‐Teller (BET) surface area increased from 5 m^2^ g^−1^ (Ni/Al_2_O_3_‐dr) to 99 m^2^ g^−1^ (Ni/Al_2_O_3_‐calc) after calcination, while a decrease from 128 m^2^ g^−1^ (Al_2_O_3_‐dr) to 91 m^2^ g^−1^ (Al_2_O_3_‐calc) was observed.

### 2.2 In Situ X‐Ray Ptychography

**Figure 3:**
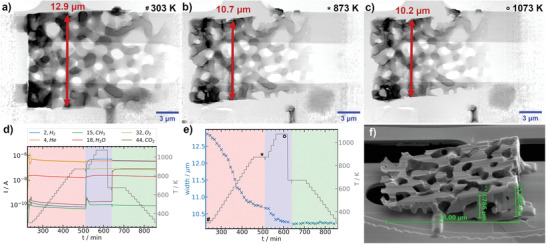
In situ XRP experiments for Ni/Al_2_O_3_. a–c) Ptychographic reconstructions with 18.6 nm pixel size, MEMS chip and windows (bright) visible in the background; a) initial state of the dried gel (“#,” 100% He); b) after calcination at 873 K (“⋆,” 20% O_2_ / He); c) after activation at 1073 K (“○,” 25% H_2_/He), total gas flow 2 mL min^−1^; d) selected MS traces and temperature (red box—calcination, blue—activation and green—reaction conditions (20% H_2_/5% CO_2_/He)); e) sample width measured at the characteristic feature as indicated by the red arrow in (a‐c) with identical conditions as in (d); f) SEM image before the experiment, the width is based on a single point measurements at each temperature at the identified characteristic feature.

The evolution of the Ni/Al_2_O_3_‐dr dried gel sample during calcination, activation, and reaction conditions was investigated by in situ XRP. This enables to follow textural changes occurring in real time, in contrast to post mortem analysis by gas sorption or Hg porosimetry. A wedge‐like structure of Ni/Al_2_O_3_‐dr was prepared (**Figure** **3**f) via focused‐ion beam (FIB) milling,^[^ ^55^ ^]^ and mounted on a micro‐electro‐mechanical systems (MEMS) Wildfire Nano‐Chip (DENSsolutions, Delft, Netherlands) by gluing using ion‐beam‐induced deposition (IBID) of a Pt precursor inside the FIB system (see Figure S2, Supporting Information). Scanning electron microscopy (SEM) images indicated sample dimensions of length ≈18 µm, width ≈13 µm and maximum thickness of ≈5 µm. The Ni/Al_2_O_3_‐dr sample on the MEMS chip was placed in a nanoreactor (see Figure S3, Supporting Information),^[^ ^53^ ^]^ which was mounted on the stage of the PtyNAMi setup at the Nanoprobe endstation of beamline P06 of the Deutsches Elektronen Synchrotron DESY (Hamburg, Germany).^[^ ^56^ ^]^ In situ XRP studies were carried out employing three different gas conditions: i) calcination (20% O_2_/He), ii) activation (25% H_2_/He), and iii) reaction (20% H_2_/5% CO_2_/He) with a constant total gas flow of 2 mL min^−1^ (see Table S3, Supporting Information). During in situ XRP the outlet gas composition was monitored by online mass spectrometry (MS) (Figure 3d). For further details on the in situ XRP experiments see Supporting Information (Figure S4, Supporting Information). Figure 3a shows the phase contrast image of the initial state of the Ni/Al_2_O_3_‐dr sample at 303 K under He atmosphere, with spatial resolution estimated to be 67.2 nm via Fourier ring correlation (FRC) (see Figure S5, Supporting Information).^[^ ^57^ ^]^ The macropore system can be readily identified, in agreement with the SEM images recorded during FIB preparation (Figure 3f and Figure S2, Supporting Information). The sample was then heated in 30 K steps up to 873 K under calcination conditions and held for ≈1 h at 873 K. At each temperature step a ptychographic projection was recorded with an acquisition time of ≈11 min under isothermal conditions, while during longer holding times multiple projections were recorded. The sample was visibly shrinking during the entire calcination period, which can be readily observed comparing the image at the final stage of the calcination (Figure 3b) with the initial state (Figure 3a). A movie of the sample changes during calcination is available in Supporting Information. The width of the sample was analyzed by aligning to a clearly identifiable feature as indicated by the arrow in Figure 3a–c, with the evolution of the width shown in Figure 3e. During calcination the width decreased from 12.9 to 10.7 µm by about 17%. The most pronounced changes occurred in the temperature range from about 400 to 800 K. The calcination was followed by activation conditions in 50 K steps up to 1073 K and a holding time of ≈1 h at 1073 K. The sample further contracted during activation, indicating continuing minor sample changes but less severe compared to those seen during calcination. The width from the initial dried gel to the activated state decreased by about 20% from 12.9 to 10.2 µm. After cooling to 673 K and switching to reaction conditions, no further significant change of sample width was observed. The observed sample changes are consistent with bulk ex situ observations during laboratory synthesis of hierarchically porous Ni/Al_2_O_3_, where monoliths were typically found to shrink about 15% in width from the dried gel to the calcined state. Despite the observed volume shrinkage, the macropore network stayed qualitatively intact during the whole in situ experiment in terms of the overall connectivity and general shape. However, no quantification is possible from only 2D projection images. During the whole in situ experiment the outlet gas was analyzed confirming the applied gas composition was present at the sample as indicated by the colored regions in Figure 3d,e. However, the amount of sample and thus evolution of gaseous products in the experiment (which would constitute an operando study) was too low to be detected via online MS analysis.

The in situ XRP studies show the potential of XRP for studying extended sample volumes (several µm) with sub‐100 nm resolution under well defined temperature and gas conditions. Comparable results from SEM or FIB‐SEM imaging are not feasible, due to insensitivity to the bulk sample or destruction of the sample, respectively. Likewise the sample volume probed here using hard X‐ray imaging is orders of magnitude larger than could be studied with transmission electron microscopy (TEM), due to electron beam attenuation. While in situ XRP studies in catalysis science were already reported before for sintering experiments on model systems,^[^ ^53^, ^58^ ^]^ we show here the application to retrieve textural information of the evolution of an applied catalyst system under different gas compositions and temperature steps. In principal the setup would also allow for missing wedge tomography acquisition, which enables to retrieve 3D quantitative information from in situ experiments.^[^ ^53^ ^]^ This is in principle analogous to TEM‐tomography, although permitting much larger sample volumes due to the low attenuation of hard X‐rays compared to electrons. However, the required time for acquisition of a limited angle tomogram in the used experimental configuration would have taken too long (≈24 h per tomogram). Despite measuring changes of the sample width, deriving further quantitative information about the pore structure is not feasible based on 2D projection data. Further PXCT experiments were therefore performed. In principle, any structural changes of the sample leading to changes in electron density can be investigated by XRP. For Ni/Al_2_O_3_ this could be exemplarily the reduction of NiO to Ni during catalyst activation. However, the achieved 2D resolution of ≈67 nm is not sufficient to directly resolve any large Ni particles which may be formed by sintering or aggregation during or after the activation step. Generally, changes in electron density can also be observed even if single individual features such as Ni particles cannot be directly resolved, as shown previously for coke deposition on a Ni/Al_2_O_3_ catalyst.^[^ ^50^ ^]^ In the present case, a quantitative analysis of the electron density is limited due to the tilting of the sample upon thermal treatment, which would require to account for changes of the sample thickness that cannot be directly obtained from only 2D projections. In summary, the ability to resolve structural changes in 2D can be challenging on extended 3D samples and is limited by the achieved 2D resolution and potential textural changes.

### 2.3 Ex Situ Ptychographic X‐Ray Computed Tomography

**Figure 4:**
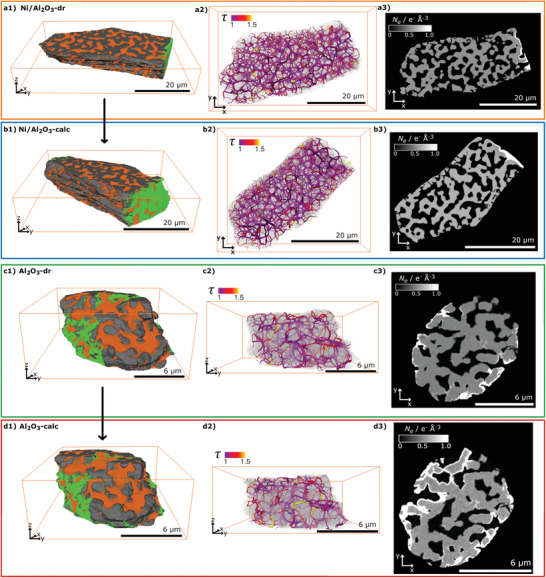
PXCT volume renderings with labeling for a1) Ni/Al_2_O_3_‐dr, b1) Ni/Al_2_O_3_‐calc, c1) Al_2_O_3_‐dr, and d1) Al_2_O_3_‐calc, showing “material” (gray), “pores” (orange), and “contamination” (green) labels, with reconstructed voxel sizes a,d) 19.9 nm and b,c) 27.9 nm. a2–d2) Pore network models derived from PXCT data, colormap representing segment tortuosity (τ), with segment size proportional to pore radius (*r*_pore_). a3–d3) Selected slices of PXCT volumes with grayscale *N*_e_ in e^−^ Å^−3^. The calcined samples b3,d3) were measured after heating the dried gels a3,c3) in air at 823 K (10 K min^−1^) for 15 min.

**Figure 5:**
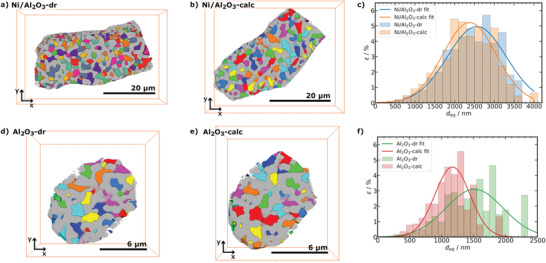
Equivalent spherical diameter (*d*_eq_) analysis of separated pore labels from PXCT. Volume renderings of “pores” (8‐bit color code) and “material” (gray) labels for a) Ni/Al_2_O_3_‐dr, b) Ni/Al_2_O_3_‐calc, d) Al_2_O_3_‐dr, and e) Al_2_O_3_‐calc. Porosity (ϵ) depending on *d*_eq_ and Gaussian fit for c) Ni/Al_2_O_3_ (*d*_eq_ binning of 200 nm, number of analyzed pores: Ni/Al_2_O_3_‐dr 971 and Ni/Al_2_O_3_‐calc 880) and f) Al_2_O_3_ (*d*_eq_ binning of 100 nm, number of analyzed pores: Al_2_O_3_‐dr 217 and Al_2_O_3_‐calc 265).

3D spatially‐resolved PXCT studies were performed to quantify changes of the pore structure during calcination of the Ni/Al_2_O_3_ and Al_2_O_3_ samples, and to recover quantitative *N*_e_ values for the hierarchically porous solids. Suitable particles of Ni/Al_2_O_3_‐dr and Al_2_O_3_‐dr were mounted on Cu tomography pins of the OMNY design^[^ ^59^ ^]^ by gluing using IBID of a Pt precursor gas inside the FIB‐SEM. The mounted particles had a diameter of ≈50 µm and ≈15 µm for Ni/Al_2_O_3_‐dr and Al_2_O_3_‐dr, respectively, as shown by SEM (Figure S6, Supporting Information). PXCT studies were performed at cSAXS beamline of the Swiss Light Source (Paul Scherrer Institut, Villigen, Switzerland) using the flOMNI endstation at an energy of 6.2 keV.^[^ ^41^, ^60^ ^]^ For experimental details see Supporting Information  and Table S4, Supporting Information. After initial PXCT scans of the dried gels, the samples were removed from the beamline and calcined in a muffle furnace. The samples on the pins were heated to 823 K with a rate of 10 K min^−1^ under flowing air with a holding time of 15 min at 823 K. It should be noted that higher temperatures during calcination were not possible due to ablation of the Cu‐pins, which happens above 873 K and would contaminate the sample. After the calcination step, comparable regions of the exact same particles were studied again by PXCT, denoted as Ni/Al_2_O_3_‐calc and Al_2_O_3_‐calc. The resulting phase contrast tomograms of Ni/Al_2_O_3_‐dr and Ni/Al_2_O_3_‐calc have voxel sizes of 19.9 and 27.9 nm and estimated resolutions by Fourier shell correlation (FSC)^[^ ^61^ ^]^ of 91.2 and 55.6 nm, respectively (Figure S7, Supporting Information). For Al_2_O_3_‐dr and Al_2_O_3_‐calc the voxel sizes are 27.9 and 19.9 nm with estimated FSC resolutions of 49.6 and 40.9 nm, respectively (Figure S8, Supporting Information). Each PXCT acquisition was performed by sequential acquisition of subtomograms. This allowed to check for and exclude potential beam damage effects, by comparing slices with neighboring projection angle recorded at time intervals of ≈20–60 min, depending on the sample (see Supporting Information). The 3D *N*_e_ distribution of the reconstructed PXCT tomograms was retrieved from the phase contrast tomograms (see Supporting Information) as described in refs. [40, 42] Selected slices of the resulting *N*_e_ tomograms are shown in **Figure** **4**a3–d3. For image analysis of all PXCT data, the whole particles were first masked from the surrounding air. Then the particle volumes were segmented by thresholding into “pores” (orange), “material” (gray), and “contamination” (green) labels based on *N*_e_ (Figure 4 a1–d1). The contamination mainly consists of Pt complexes deposited during FIB preparation, or from redeposition of a mixture of materials (sample, Cu pin) which can occur during ion beam milling. These contaminants were easily identified and excluded from further analysis of porosity. The obtained “pore” labels of all samples were additionally transformed into global skeleton networks, which are used as pore network models as shown in Figure 4 a2–d2. Additionally, the “pore” labels were separated into individual pores as shown in **Figure** **5**a,b,d,e with an 8‐bit color code for Ni/Al_2_O_3_‐dr, Ni/Al_2_O_3_‐calc, Al_2_O_3_‐dr, and Al_2_O_3_‐calc, respectively. For details regarding image analysis and processing steps see Supporting Information. The different labels and pore network models were used to quantify the pore structure of the samples.

### 2.4 3D Pore Structure Characterization by PXCT Image Analysis

A connected macropore network can be directly identified in all four tomography datasets (Figure 4). The spatial resolution (≈50 to 90 nm) of the tomograms is not sufficient to directly resolve mesopores within the samples,^[^ ^36^, ^52^, ^54^ ^]^ however PXCT can quantify such features indirectly by *N*_e_ analysis discussed in the following section. The presence of mesopores in the calcined samples was expected from Hg porosimetry shown above, while a hierarchical pore system was also found previously for an activated Ni/Al_2_O_3_ catalyst studied by correlative multi‐scale PXCT and electron tomography.^[^ ^36^ ^]^ Quantitative label analysis of the “pore” and “material” labels (Figure 4 a1–d1)) allowed to determine the label volumes and to calculate the macroporosity (ϵ_M_) of the measured particle volumes (see Supporting Information  for details). The porosity analysis results are summarized in **Table** **1** for all samples. The determined ϵ_M_ for Ni/Al_2_O_3_‐dr showed only a minor increase after calcination to Ni/Al_2_O_3_‐calc, while both states showed comparable macroporosity as in a previous PXCT study of the same material after activation (ϵ_M_ = 46.3%).^[^ ^36^ ^]^ For Al_2_O_3_‐dr the ϵ_M_ slightly decreased after calcination to Al_2_O_3_‐calc. For both catalyst types, the *N*_e_ slices of similar particle regions showed significant volume shrinkage comparable to the in situ XRP experiments (Figure 4 a3–d3, note difference in scale bars).

**Table 1:** Summary of pore characterization based on PXCT data, including: macroporosity (ϵ_M_), mesoporosity (ϵ_m_), total porosity (ϵ_tot_), mean equivalent spherical pore diameter of separated pores (${\bar{d}}_{\text{eq}}$), mean pore diameter of skeleton‐based pore network model (${\bar{d}}_{\text{pore}}$), mean length of skeleton segments (${\bar{l}}_{\text{cu,pore}}$), mean tortuosity of skeleton segments ($\bar{\tau }$), mean coordination number of all nodes in skeleton network ($\bar{CN}$), mean coordination number of branching nodes in skeleton network (${\bar{CN}}_{\text{br}}$)

	Ni/Al_2_O_3_		Al_2_O_3_	
	Dried	Calcined	Dried	Calcined
ϵ_M_/%	43.9	44.5	34.9	33.6
ϵ_m_/%	29.4	23.4	34.3	30.1
ϵ_tot_/%	73.3	67.9	69.2	63.7
${\bar{d}}_{\text{eq}}$/µm	2.54 ± 0.65	2.43 ± 0.66	1.54 ± 0.41	1.19 ± 0.31
${\bar{d}}_{\text{pore}}$/µm	1.36 ± 0.44	1.17 ± 0.39	0.57 ± 0.20	0.47 ± 0.17
${\bar{l}}_{\text{cu,pore}}$/µm	1.64 ± 1.02	1.51 ± 0.83	1.03 ± 0.55	0.85 ± 0.49
$\bar{\tau }$/1	1.1 ± 0.1	1.1 ± 0.3	1.1 ± 0.1	1.1 ± 0.1
$\bar{CN}$/1	2.3 ± 1.0	2.6 ± 1.0	2.6 ± 1.0	2.4 ± 1.0
${\bar{CN}}_{\text{br}}$/1	3.1 ± 0.3	3.1 ± 0.4	3.1 ± 0.4	3.1 ± 0.3

See Supporting Information for definition, calculation, and sensitivity of all measures.

The macropore structure can be further characterized by the equivalent spherical pore diameter (*d*_eq_, see Equation (S10), Supporting Information) of the individually labeled pores (Figure 5). The porosity‐weighted *d*_eq_ distribution for Ni/Al_2_O_3_ and Al_2_O_3_ are shown in Figure 5c,f, respectively. From these distributions, the mean ${\bar{d}}_{\text{eq}}$ was extracted (Table 1). The ${\bar{d}}_{\text{eq}}$ slightly decreased from Ni/Al_2_O_3_‐dr to Ni/Al_2_O_3_‐calc after calcination, showing comparable values to an activated catalyst studied previously (${\bar{d}}_{\text{eq}}$ = 2.55 ± 0.2 µm).^[^ ^36^ ^]^ For Al_2_O_3_, the decrease of ${\bar{d}}_{\text{eq}}$ was more pronounced after calcination, also clearly visible in the *d*_eq_ distribution (Figure 5f).

**Figure 6:**
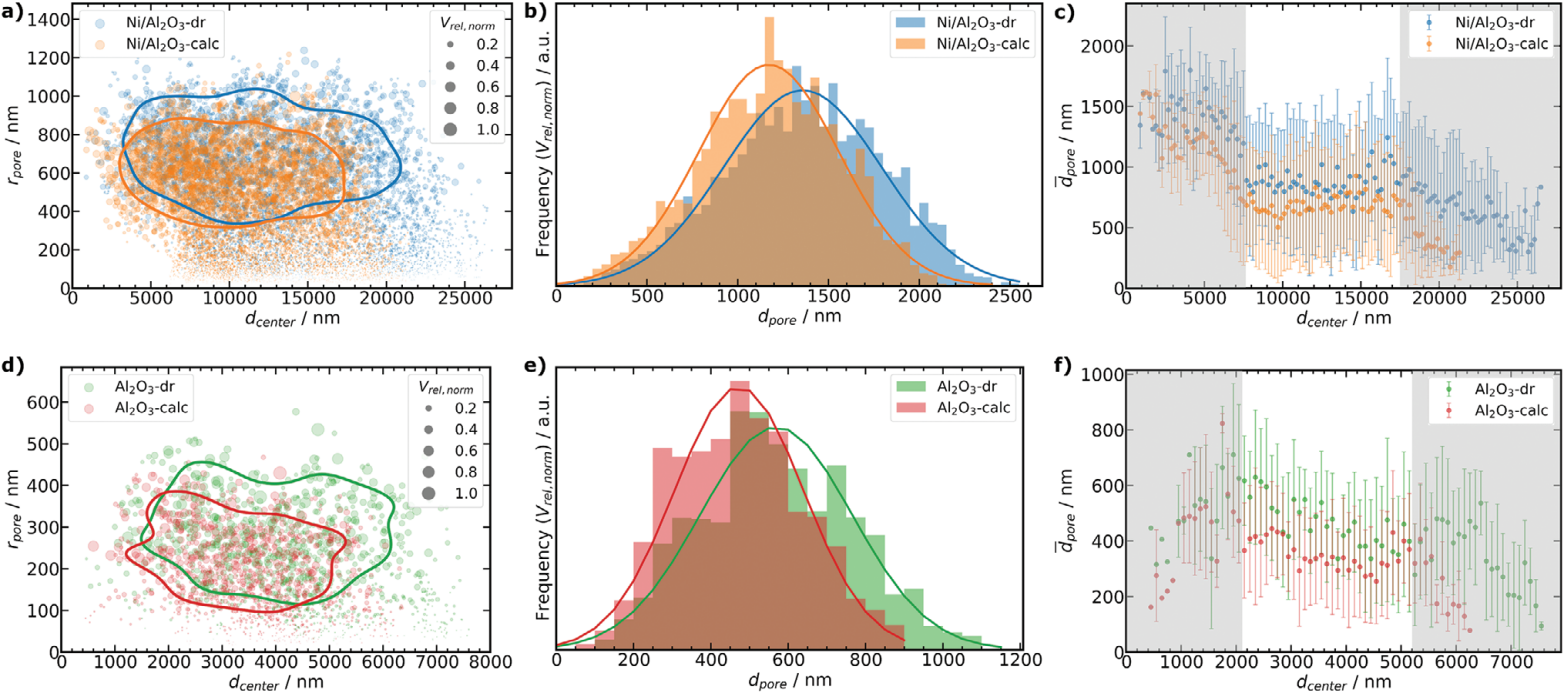
Characterization based on pore network models in Figure 4 a2–d2. a,d) pore radius (*r*_pore_) depending on distance to the particle center (*d*_center_), with scatter size showing relative normalized pore volume (*V*_rel, norm_) for Ni/Al_2_O_3_ and Al_2_O_3_, respectively. Contours represent the *V*_rel, norm_ weighted probability to enclose 75% of all pores (see Supporting Information for details, number of segments as pores: Ni/Al_2_O_3_‐dr 6415, Ni/Al_2_O_3_‐calc 5654, Al_2_O_3_‐dr 1078, and Al_2_O_3_‐calc 1119). b,e) *V*_rel, norm_ weighted pore diameter (*d*_pore_) distribution for Ni/Al_2_O_3_ and Al_2_O_3_, respectively. c,f) Dependence of mean pore diameter (${\bar{d}}_{\text{pore}}$) on *d*_center_ for Ni/Al_2_O_3_ (*d*_center_ binning 200 nm) and Al_2_O_3_ (*d*_center_ binning 100 nm), respectively. Data is shown as mean values with standard deviation as errorbars. Shaded regions indicate low statistical relevance defined by qualitative inspection of the volumes (surface and core artifacts).

As *d*_eq_ is only a strictly correct assumption for spherical pores and in our case rather represents the pore body of the macropores, the macropore shape and structure were further characterized using a skeleton based pore network model (see Supporting Information for details). The retrieved pore network models consist of connected spatial graphs, constructed from pore nodes connected to each other via pore segments. The pore network models for Ni/Al_2_O_3_‐dr, Ni/Al_2_O_3_‐calc, Al_2_O_3_‐dr, and Al_2_O_3_‐calc are plotted in (Figure 4 a2–d2). A single primary spatial graph (i.e., a fully connected network of pore segments) was identified for all samples, which confirms the presence of a well‐connected macropore structure in each case. Further analysis was restricted to this main graph, therefore excluding any outlying or disconnected features, for example, at the external particle boundary. For each pore segment, a cylindrical macropore radius/diameter (*r*_pore_/*d*_pore_), segment volume, segment tortuosity (τ), and segment length (*l*_cu, pore_) can be retrieved. For each node, the location in the particle, coordination number, and thus amount of terminating and branching nodes, can be analyzed. The location of nodes and segments is defined here by the distance to the gravimetric center of the particle volume (*d*_center_), while the segment coordinates are approximated as the center point of the connected nodes. Statistical analysis results of the pore network models are shown in **Figure** **6**a–c for Ni/Al_2_O_3_ and Figure 6d–f for Al_2_O_3_, respectively. Figure 6a,d shows the *r*_pore_ distribution of each segment relative to *d*_center_ for Ni/Al_2_O_3_ and Al_2_O_3_, with scatter sizes indicating normalized relative segment volume (*V*_rel, norm_). For both samples, calcination generally led to a decrease in the macropore radii (*r*_pore_) and appeared to indicate particle volume shrinkage, with a higher density of segments observed at lower *d*_center_ values after calcination. The significance of the segment *r*_pore_ distribution was tested by performing a kernel density estimation (KDE) of the volume weighted 2D histograms (Figures S12 and S13, Supporting Information) as implemented in the python seaborn package.^[^ ^62^ ^]^ The resulting KDE‐contours enclose 75% of the volume weighted segments highlighting differences between the dried gel and calcined states. The decrease in macropore radii is additionally highlighted by the *V*_rel, norm_ weighted distribution of *d*_pore_ shown in Figure 6b,e for Ni/Al_2_O_3_ and Al_2_O_3_. The mean macropore diameter (${\bar{d}}_{\text{pore}}$) calculated from the spatial graphs therefore also decreased after calcination for both catalysts as expected (Table 1). Furthermore, the volume shrinkage observed previously during the in situ XRP studies and on visual inspection of the PXCT data was also confirmed (Figure 6a,d). In addition to the overall *d*_pore_ distribution, Figure 6c,f highlights the dependence of ${\bar{d}}_{\text{pore}}$ on *d*_center_ for each catalyst system. Small and large *d*_center_ values indicated by the shaded region in Figure 6c,f, were not considered statistically relevant due to limited sampling volume, and likelihood of anomalous values at the external particle boundary. In general and particularly for Ni/Al_2_O_3_, a quite homogeneous ${\bar{d}}_{\text{pore}}$ distribution was observed independent of position within the particle as defined by *d*_center_. This further supports the presence of a homogeneous and well‐ordered macropore structure in each sample. The distribution of ${\bar{d}}_{\text{pore}}$ relative to *d*_center_ did not change significantly following calcination.

In addition to quantification of porosity characteristics, further information is readily available from tomographic image analysis, which cannot be directly retrieved from N_2_ sorption or Hg porosimetry analysis. For example, segment tortuosity (τ) and coordination number of the nodes (*CN*) can be derived (Table 1). Here the mean coordination number $\bar{CN}$ of the nodes was between 2.3 to 2.6 for all samples. As these values are strongly influenced by terminal nodes at the particle exterior, the mean coordination number including only branched nodes (${\bar{CN}}_{\text{br}}$) is also reported (Table 1), which is more sensitive to potential internal changes of the pore structure compared to $\bar{CN}$. ${\bar{CN}}_{br}$ was 3.1 for all four samples. The average segment tortuosity ($\bar{\tau }$) was 1.1 for all four samples. As the segments are short compared to the sample volume investigated by PXCT, $\bar{\tau }$ should not be mistaken for macroscopic tortuosity, which is typically employed for diffusion calculations.^[^ ^63^ ^]^ However, τ can be used to search for specific regions in the pore network that might form a bottleneck for mass transport in such highly connected pore structures. Therefore, $\bar{CN}$ and $\bar{\tau }$ were analyzed regarding their dependence on *d*_center_ as shown in Figures S9 and S10, Supporting Information, respectively. No significant changes were observed for either catalyst system between dried gel and calcined states. This supports the results from the overall mean values of $\bar{CN}$, ${\bar{CN}}_{\text{br}}$, and $\bar{\tau }$ confirming a homogeneous macropore structure. Furthermore, this quantitatively confirms the observations made during the in situ XRP experiments, indicating no obvious pore opening, closing, or disruption of the macropore network.

### 2.5 Electron Density Analysis of PXCT Data

**Figure 7:**
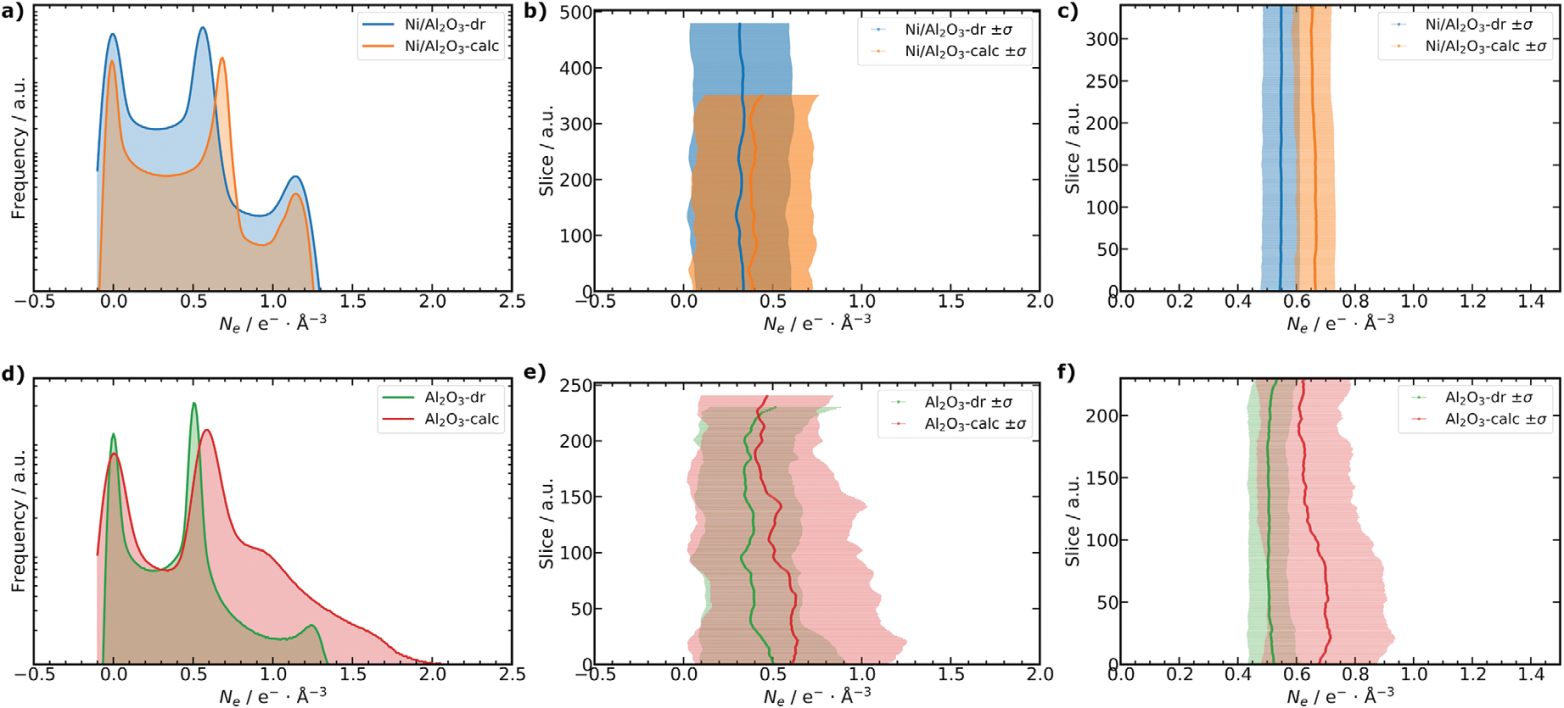
Electron density (*N*_e_) analysis of PXCT data. a,d) *N*_e_ distribution for the whole particle volumes of Ni/Al_2_O_3_ and Al_2_O_3_, respectively. b,e) Slice dependent analysis of the mean *N*_e_ with standard deviation (σ) as errorbars for the whole particle volume of Ni/Al_2_O_3_ and Al_2_O_3_, respectively. c,f) Selected slice range analysis of the mean *N*_e_ with σ as errorbars only for the segmented “material” label of the nanoporous solid for Ni/Al_2_O_3_ and Al_2_O_3_, respectively.

Direct quantification of the pore structure based on image analysis is generally restricted to the spatial resolution of the measurement. Here, PXCT offered sufficient spatial resolution (≈50 to ≈90 nm) for detailed analysis of the majority of macropores. On the other hand, direct quantification of mesopore features below the spatial resolution limit was neither feasible nor statistically meaningful. However, an advantage of PXCT compared to other tomographic imaging methods is that quantitative information about the local *N*_e_ can be obtained, which is indicative of the material composition at each sampling point regardless of the exact spatial resolution.^[^ ^39^, ^40^, ^41^, ^42^ ^]^ The overall *N*_e_ histogram of the whole particle volumes measured by PXCT is shown in **Figure** **7**a,d for Ni/Al_2_O_3_ and Al_2_O_3_, respectively. Three main features were observed and labeled, corresponding to the image segmentation procedure discussed previously: *N*_e_ ≈ 0 e^−^ Å^−3^ as air/macropores (“pores” label), *N*_e_ ≈ 0.5 to 0.55 e^−^ Å^−3^ as nanoporous material (“material” label), and *N*_*e*_ > 0.9 e^−^ Å^−3^ labeled as “contamination” from sample preparation. Already in the overall *N*_e_ distribution (Figure 7a,d), and individual *N*_e_ slices (Figure 4a3–d3) of the whole particle volume, a significant shift of the “material” label peak to higher values of ≈ 0.6 to 0.65 e^−^ Å^−3^ was observed after calcination of the dried gels for both catalyst systems. This may indicate a densification of the nanoporous material, also supported by an increase of the skeletal density (ρ_skel_) observed by He pycnometry after calcination of the dried gel samples (Table S1, Supporting Information). Figure 7b,e shows the slice dependent analysis of the mean electron density (${\bar{N}}_{e}$) of the whole particle volume for Ni/Al_2_O_3_ and Al_2_O_3_, respectively. Slice dependent analysis allows for easy identification and exclusion of regions with strong contamination from further analysis.^[^ ^50^ ^]^ For the segmented “material” label, the overall *N*_e_ distribution is shown in Figure S16, Supporting Information  and the slice dependent analysis of ${\bar{N}}_{e}$ in Figure 7c,f for Ni/Al_2_O_3_ and Al_2_O_3_, respectively. For Ni/Al_2_O_3_, a homogeneous and significant shift of ${\bar{N}}_{e}$ is found for the selected slice range (Figure 7c). In the case of Al_2_O_3_, a similar significant shift of ${\bar{N}}_{e}$ was observed (Figure 7f), while the ${\bar{N}}_{e}$ of Al_2_O_3_‐calc was not as homogeneous as for Ni/Al_2_O_3_‐calc. This can be partly attributed to the smaller sample volume of the Al_2_O_3_ particle compared to Ni/Al_2_O_3_ and thus stronger influence of the surface structure accompanied with surface defects and artifacts. Furthermore, some increased formation of contaminant species was observed after calcination of Al_2_O_3_ (Figure 4 d3) probably originating from the tip of the Cu sample pin, which was not found for Ni/Al_2_O_3_. Nevertheless, both systems confirm an increase in *N*_*e*_ for the “material” label, corresponding to a densification of the nanoporous solid after calcination.

With prior knowledge about the chemical composition, the observed *N*_e_ can be converted into observed mass density (ρ),^[^ ^40^, ^42^ ^]^ in order to indirectly measure porosity even below the spatial resolution limit of the measurement. A detailed description of all porosity calculations and derivation is given in the Supporting Information. The porosity of the “material” label (ϵ_mat_) can be derived by comparing the observed (ρ) and theoretical mass density (ρ_theo_). Subsequently, ϵ_mat_ was used to calculate the mesoporosity (ϵ_m_) and total porosity (ϵ_tot_) of the samples, with ϵ_tot_ as the sum of ϵ_M_ and ϵ_m_. The theoretical density of the structures can be obtained from a known crystal structure or approximated experimentally as ρ_skel_ by He pycnometry (see Table S5, Supporting Information). For Al_2_O_3_, ρ_theo_ was taken as the X‐ray density of a reported γ‐Al_2_O_3_ structure,^[^ ^64^ ^]^ and for Ni/Al_2_O_3_ ρ_theo_ was defined as ρ_skel_ determined experimentally by He pycnometry. The mode of the *N*_e_ distribution of the “material” label (see Table S5, Supporting Information) was then used to calculate ϵ_mat_, from which ϵ_m_ and ϵ_tot_ were derived (Table 1). An exact chemical composition of the dried gel materials Al_2_O_3_‐dr and Ni/Al_2_O_3_‐dr is unknown, as these probably still contain organic and solvent residues as shown by CHN‐analysis. For simplification and in order to calculate ρ, the chemical composition of the dried gels was approximated by the known structures of the calcined samples (Ni_0.43_Al_2_O_4_, γ‐Al_2_O_3_).^[^ ^36^, ^52^, ^54^ ^]^ It is therefore important to note that only for the calcined states do the above porosity values correspond to true porosity. Since ϵ_mat_, ϵ_m_, and ϵ_tot_ are calculated based on sub‐resolution information, that is, local *N*_e_ of the material label, therefore the porosity values of the dried gels are influenced by organic and solvent residues, potential intermediate Al_2_O_3_ and Ni/Al_2_O_3_ structures or blocked pores which cannot be directly resolved. In this respect, mesoporosity values are described as an “apparent” ϵ_m_ for the dried gels, which can only be carefully compared to conventional pore characterization methods. In more detail, the “apparent” ϵ_m_ for the dried gels from PXCT can hardly resolve or distinguish between accessible or inaccessible porosity, as it is a sub‐resolution information. This is contrary to conventional methods, that is, Hg porosimetry and N_2_ sorption, which indeed can only resolve accessible pores to the respective probe species (Hg or N_2_). Therefore, ϵ_m_ from PXCT experiments and conventional methods can only be strictly compared for the calcined samples. In the calcined samples, ϵ_m_ is not influenced anymore by solvent or organic residues, which might fill the mesopores in the dried gel state making them inaccessible for conventional methods. Nevertheless, this information provides valuable and potentially quantitative insights into sample changes occurring during calcination. On calcination of Ni/Al_2_O_3_‐dr to form Ni/Al_2_O_3_‐calc, ϵ_m_ decreased from 29.4% to 23.4% and ϵ_tot_ from 73.3% to 67.9%. A similar trend was also observed for Al_2_O_3_‐dr to Al_2_O_3_‐calc, where ϵ_m_ decreased from 34.3% to 30.1% and ϵ_tot_ from 69.2% to 63.7%. Thus, during calcination a significant densification of the nanoporous solid occurs for both systems. ϵ_m_ of Ni/Al_2_O_3_‐calc is well in line with the value of 24.1% previously reported by electron tomography, where direct imaging of mesopores is feasible due to much higher spatial resolution than PXCT.^[^ ^36^ ^]^ It is important to note however, that electron tomography is restricted to samples with several orders of magnitude smaller volume than can be measured with hard X‐ray tomography. Particularly for hierarchically porous or complex catalysts, X‐ray microscopy potentially provides a more representative view of the sample structure. The derived ϵ_m_ is also in agreement with previous Hg porosimetry combined with He pycnometry.^[^ ^36^ ^]^ However, conventional porosimetry techniques probe only the bulk sample and cannot provide spatially‐resolved data comparable to that produced by tomography. The agreement with electron tomography and Hg porosimetry therefore validates the quantitative *N*_e_ based porosity analysis enabled here by PXCT, even if the resolution is currently not high enough to directly visualize mesopores in the sample.

### 2.6 Pore Structure Evolution during Calcination

The deconvolution of different processes occurring during synthesis and treatment of hierarchical materials (e.g., by calcination) and their influence on the final pore structure is challenging to unravel. In previous work, Tokudome et al. proposed that mesopores were generated during calcination by crystallization of alumina, as they were not detected by Hg porosimetry in the dried gels.^[^ ^54^ ^]^ In the current study, the presence of macropores in Al_2_O_3_‐dr and Ni/Al_2_O_3_‐dr was found by Hg porosimetry, which was shown by PXCT, XRP, and SEM imaging to be an ordered and connected macropore network. While the resolution of PXCT obtained here was not sufficient to study mesopores directly, *N*_e_ analysis revealed an apparent ϵ_m_ of about 29% and 34% for Al_2_O_3_‐dr and Ni/Al_2_O_3_‐dr, respectively. From these results it may be concluded that the mesopore system is probably already preorganized in the dried gels, but filled with solvent or polymer species, while exact knowledge about the precursors oxide structure is missing. During calcination, PXCT and in situ XRP revealed an extensive overall volume shrinkage accompanied with a densification of the nanoporous solid, which is also in line with observations during calcination in the laboratory synthesis. While in situ XRP could monitor the changes occurring in real time under controlled gas and temperature conditions, PXCT was required to quantify these changes in terms of pore structure. Based on PXCT, the sample macroporosity was almost constant during calcination, while the mesoporosity and total porosity decreased for both systems (see Table 1). A decrease was also observed for both *d*_eq_ and *d*_pore_ after calcination based on PXCT. *w*_P, M, Hg_ obtained from Hg porosimetry showed the same trend for Ni/Al_2_O_3_ and a similar sample of Al_2_O_3_ compared to the imaging studies. N_2_ sorption analysis of Al_2_O_3_‐dr revealed presence of mesopores in the dried gel, while a mild thermal treatment at 373 K was carried out before the experiment. The heat treatment can be sufficient to already remove solvent or H_2_O from the dried gel leading to changes of the sample. For Ni/Al_2_O_3_‐dr no significant mesopores were identified by N_2_ sorption. This obviously illustrates a major drawback of conventional methods compared to PXCT in studying sol–gel materials in the intermediate stages of synthesis. While in PXCT such method related changes can be identified by analysis of the acquired subtomograms, which revealed some X‐ray beam induced sample changes for the dried gel samples in the beginning of the measurement as detailed in Supporting Information. The advantages of X‐ray tomography based pore characterization compared to Hg porosimetry to derive quantitative pore structure descriptors has been reported in several studies.^[^ ^30^, ^31^, ^36^, ^65^, ^66^, ^67^, ^68^ ^]^ In the present work ${\bar{CN}}_{\text{br}}$ is 3.1 for all four studied samples, from which it can be concluded that during calcination no major changes of the connectivity of the macropore network occur. This is furthermore supported by the analysis of the segment τ and that for all PXCT data sets only one major spatial graph was identified to represent the pore network. The scanning of the same particles of Al_2_O_3_ and Ni/Al_2_O_3_ as dried gels and after calcination of these gels allows to directly compare the results for both states. This eliminates the challenge of selecting a representative sample particle, which is a common problem for imaging methods. Changes of macropores upon thermal treatment investigated by X‐ray microtomography have been reported in literature.^[^ ^69^, ^70^, ^71^ ^]^ However, the resolution of X‐ray microtomography can only cover larger macropores and not all relevant macropore length scales. PXCT as shown in this study enables to routinely study materials with sub 50 nm resolution on tens of µm sample sizes, in the current implementation.

The experimental strategy shown here can be generally employed to study textural and structural changes of materials with high spatial resolution, for example during thermal treatment as shown here. In situ XRP can be currently performed in 2D experiments, while a future extension into 3D studies is feasible. This allows to actually follow the evolution of porous materials during their synthesis and to elucidate the influence of different process steps that are typically required. The method is not only limited to the sol–gel strategy discussed in this work, but can be readily extended to templating, casting or other self‐assembly strategies which are common in literature.^[^ ^5^, ^7^, ^10^, ^11^, ^12^, ^13^, ^14^, ^15^, ^16^, ^17^, ^18^, ^19^ ^]^ On the other hand, a combination with ex situ PXCT allows to directly retrieve quantitative 3D spatially‐resolved textural and structural information about different states of the synthesis. Additionally, combining X‐ray ptychography with other contrast modes, like X‐ray fluorescence, X‐ray diffraction or X‐ray absorption spectroscopy can lead to further complementary insights into the studied material. Comprehensively, in situ XRP and PXCT provide the opportunity to develop fundamental understanding of complex synthesis procedures, which can be generally applied to understand the synthesis of functional materials, specifically hierarchically porous structures in the present case.

## 3 Conclusion

This study details the pore structure evolution of sol–gel synthesized Ni/Al_2_O_3_ and Al_2_O_3_ materials during calcination from dried gels. The calcined materials exhibit a hierarchical structure of meso‐ and macropores, which can improve the mass transport properties and overall performance in catalytic applications.^[^ ^3^, ^4^, ^5^, ^6^, ^7^, ^36^, ^52^ ^]^ However, a detailed understanding of the complex synthesis procedure to tailor such pore structures is elusive and cannot be easily determined by conventional pore characterization techniques, that is, gas sorption and Hg porosimetry. Here, hard X‐ray ptychography and ptychographic tomography are highlighted as enabling techniques to study textural and structural changes during calcination, achieving high spatial resolution down to 50 nm on extended sample volumes up to 50 µm in diameter. From the dried gel to the calcined samples the macropore diameter decreases significantly for both systems, Ni/Al_2_O_3_ and Al_2_O_3_. In addition to direct imaging covering almost the entire macropore length scale, PXCT can quantify and track the evolution of local electron density in the nanoporous solid. The electron density increases upon calcination showing a densification of the nanoporous solid, which leads to a decrease of the apparent mesoporosity during calcination. However, as the structure of the dried gels is not known exactly, further targeted in situ studies such as X‐ray total scattering or X‐ray absorption spectroscopy during calcination could be used to further differentiate the influence of the precursor oxide structure and polymer, solvent or H_2_O. With continuing progress in the development of fourth generation or diffraction‐limited synchrotron sources, PXCT is expected to routinely reach sub 10 nm resolution on similar sample volumes as in the present study, and to permit more rapid acquisition of tomograms than shown here.^[^ ^39^, ^72^, ^73^ ^]^ This would enable to directly study both larger mesopores and macropores over extended sample volumes, potentially also in a high throughput manner. Hard X‐ray ptychography is emerging as a key technique for understanding textural and structural changes in the synthesis of hierarchically porous materials, with applications in heterogeneous catalysis, adsorbents, membranes and numerous other fields.

## Conflict of Interest

The authors declare no conflict of interest.

## Supporting information

Supporting Information

Supplemental Movie 1

## Data Availability

The data that support the findings of this study are openly available in KITopen at https://doi.org/10.5445/IR/1000140344, reference number 1000140344.
