# Supplementary material for: Evolution of Hierarchically Porous Nickel Alumina Catalysts Studied by X‐Ray Ptychography
Source: Adv Sci (Weinh). 2022 Jan 20;9(8):2105432. doi: 10.1002/advs.202105432 (PMC8922122; doi:10.1002/advs.202105432)
Supplement: Supplementary file 1 — Supporting Information [file ADVS-9-2105432-s001.pdf]

ADVANCED SCIENCE
Open Access

Supporting Information

for *Adv. Sci.*, DOI 10.1002/advs.202105432

Evolution of Hierarchically Porous Nickel Alumina Catalysts Studied by X-Ray Ptychography

*Sebastian Weber, Ana Diaz, Mirko Holler, Andreas Schropp, Mikhail Lyubomirskiy, Ken L. Abel, Maik Kahnt, Arno Jeromin, Satishkumar Kulkarni, Thomas F. Keller, Roger Gläser and Thomas L. Sheppard**

ADVANCED SCIENCE
Open Access

# Supporting Information

for *Adv. Sci.,* DOI: 10.1002/advs.202105432

# Evolution of Hierarchically Porous Nickel Alumina Catalysts Studied by X-ray Ptychography

*Sebastian Weber, Ana Diaz, Mirko Holler, Andreas Schropp, Mikhail Lyubomirskiy, Ken L. Abel, Maik Kahnt, Arno Jeromin, Satishkumar Kulkarni, Thomas F. Keller, Roger Glaser and Thomas L. Sheppard**

# Evolution of Hierarchically Porous Nickel Alumina Catalysts Studied by X-ray Ptychography

Sebastian Weber, Ana Diaz, Mirko Holler, Andreas Schropp, Mikhail Lyubomirskiy, Ken L. Abel, Maik Kahnt, Arno Jeromin, Satishkumar Kulkarni, Thomas F. Keller, Roger Gläser and Thomas L. Sheppard*

S. Weber, Dr. T. L. Sheppard
Institute for Chemical Technology and Polymer Chemistry, Karlsruhe Institute of Technology (KIT), Engesserstr. 20, 76131 Karlsruhe, Germany
Institute of Catalysis Research and Technology, Karlsruhe Institute of Technology (KIT), Hermann-von-Helmholtz-Platz 1, 76344 Eggenstein-Leopoldshafen, Germany
Email Address: thomas.sheppard@kit.edu

Dr. A. Diaz, Dr. M. Holler
Paul Scherrer Institut, 5232 Villigen PSI, Switzerland

Dr. A. Schropp, Dr. M. Lyubomirskiy, A. Jeromin, S. Kulkarni, Dr. T. F. Keller
Deutsches Elektronen-Synchrotron DESY, Notkestrasse 85, 22607 Hamburg, Germany

K. L. Abel, Prof. Dr. R. Gläser
Institute of Chemical Technology, Universität Leipzig, Linnéstraße 3, 04103 Leipzig, Germany

Dr. M. Kahnt
MAX IV Laboratory, Fotongatan 2,225 94 Lund, Sweden

A. Jeromin, S. Kulkarni, Dr. T. F. Keller
Centre for X-ray and Nano Science (CXNS), Deutsches Elektronen-Synchrotron DESY, Notkestrasse 85, 22607 Hamburg, Germany

Dr. T. F. Keller
Physics Department, University of Hamburg, 20355 Hamburg, Germany

# Contents

1 Catalyst synthesis 3

2 General characterization 4
2.1 Nitrogen sorption . . . 4
2.2 Mercury porosimetry . . . 4
2.3 Helium pycnometry . . . 4
2.4 Elemental analysis . . . 5

3 *In situ* X-ray ptychography (XRP) 6
3.1 Sample preparation . . . 6
3.2 Synchrotron experiments . . . 6
3.3 Ptychographic reconstructions and image analysis . . . 8

4 *Ex situ* ptychographic X-ray computed tomography (PXCT) 9
4.1 Sample preparation . . . 9
4.2 Synchrotron experiments . . . 9
4.3 Ptychographic and tomographic reconstruction . . . 10
4.4 Image analysis . . . 11
4.5 Pore system characterization . . . 12
4.6 Electron density analysis . . . 16

5 Statistical Analysis 18

# 1 Catalyst synthesis

The samples studied in this work were synthesized by an adapted sol-gel method as reported in literature.[1,2] Briefly, the samples were synthesized as following.

For the Ni/$Al_2O_3$-dr sample, 12.53 g $AlCl_3 \cdot 6\,H_2O$ (99 %, AlfaAesar), 2.88 g $NiCl_2 \cdot 6\,H_2O$ (98 %, AlfaAesar), and 0.200 g polyethylene oxide (PEO, molecular weight 900,000, no purity given, AlfaAesar) were dissolved in an EtOH-$H_2O$-mixture (absolute, VWR) using a polypropylene container (VWR, $V = 60\,\mathrm{cm}^{-3}$, $d = 33\,\mathrm{mm}$, $h = 70\,\mathrm{mm}$). The resulting molar ratio of $n_{\mathrm{M}} / n_{\mathrm{EtOH}} / n_{\mathrm{H_2O}} / n_{\mathrm{PEO}}$ is $1 / 5.4 / 30.1 / 3.5 \cdot 10^{-6}$ ($n_{\mathrm{M}} = n_{\mathrm{Ni}} + n_{\mathrm{Al}}$) with a targeted Ni content of 20 wt.%. The resulting solution was cooled to 274-276 K using an ice bath. Afterwards, 11.52 g of propylene oxide (PO, 99.5 %, Acros Organics) were added rapidly while vigorous stirring with 600 rpm. The $n_{\mathrm{M}}/n_{\mathrm{PO}}$ ratio was 3.1. After stirring the mixture for 3 min in the ice bath, it was further stirred for 7 min at room temperature. Subsequently, the container was sealed and kept for 24 h at 323-328 K. Afterwards, the resulting gel was kept in a solvent exchange bath of EtOH (99.5 % denaturated with 1 % butan-2-one, VWR) for 7 d. Finally, the sample was dried at room temperature to obtain the dried gel sample.

For the $Al_2O_3$-dr sample, the same procedure was applied with the following amounts of chemicals: 7.726 g $AlCl_3 \cdot 6\,H_2O$, 0.1613 g PEO, 5.762 g of PO. The resulting molar ratio of $n_{\mathrm{M}} / n_{\mathrm{EtOH}} / n_{\mathrm{H_2O}} / n_{\mathrm{PEO}}$ is $1 / 5.4 / 30.1 / 5.6 \cdot 10^{-6}$ and $n_{\mathrm{M}}/n_{\mathrm{PO}}$ ratio was 3.1.

The samples Ni/$Al_2O_3$-dr and $Al_2O_3$-dr were furthermore calcined at 1223 K (ambient air) for 12 h ($2\,\mathrm{K\,min^{-1}}$). These calcined samples are used as reference in the general characterization studies.

# 2 General characterization

## 2.1 Nitrogen sorption

$N_2$ sorption studies were performed with a Sorptomatic 1990 (Porotec) for Ni/$Al_2O_3$ samples and on a BELSORP-miniX (MICROTRAC MRB) for the other samples. Calcined samples were degassed for at least 4 h at 523 K, while the dried gel samples were treated at at 373 K, $< 10$ Pa for $> 48$ h. The treatment of the dried gels before $N_2$ sorption leads to a mass loss of $\approx$20 %. The adsorption and desorption isotherms were recorded at 77 K and analyzed using the ASiQwin software (Quantachrome Instruments) for Ni/$Al_2O_3$ and BELMaster software (MICROTRAC MRB) for other samples. The total pore volume ($V_{P,tot,N_2}$) was determined at $p/p_0 \geq 0.95$. The specific surface area ($A_{BET}$) was determined by analysis of the adsorption branch with the BET method ($p/p_0 = 0.05 - 0.03$).[3] The pore width distribution with the average pore width ($w_{P,N_2}$) was obtained from the desorption branch by the BJH method.[4]

## 2.2 Mercury porosimetry

Hg porosimetry studies were performed with a Pascal 140 (ThermoScientific) for pressures up to 0.25 MPa and a Pascal 440 (ThermoScientific, Waltham, MA, USA) for pressures between 0.25 MPa to 400 MPa. The Ni/$Al_2O_3$-dr sample was measured after drying without any further treatment. $Al_2O_3$-dr was studied by $N_2$ sorption before Hg porosimetry, therefore the sample observed a heat treatment as described above. The pore width ($w_{P,Hg}$) was calculated by the Washburn equation[5] using a contact angle of 140 ° and a surface tension of 0.48 N m$^{-1}$. The maximum differential pore volume was used to determine the modal mesopore width ($w_{P,m,Hg}$) and modal macropore width ($w_{P,M,Hg}$). The pore width distribution for the dried gel and calcined samples of Ni/$Al_2O_3$ and $Al_2O_3$ are shown in Figure S 1. For the $Al_2O_3$ system, the comparison of the dried gel and calcined state was investigated on a different sample batch compared to the one used for imaging studies.

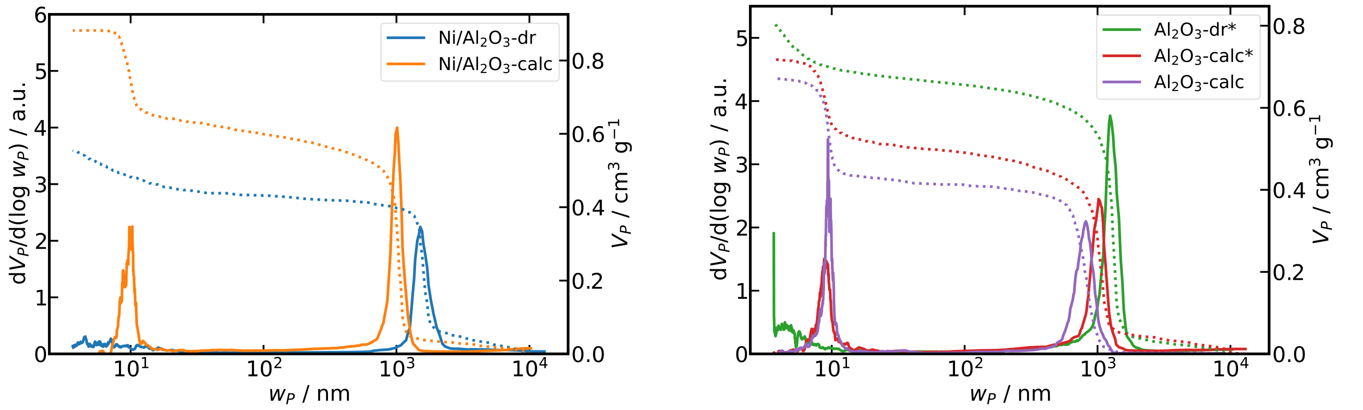


**Figure S 1** Hg porosimetry data for the Ni/$Al_2O_3$ (left) and $Al_2O_3$ (right) samples as dried gel and calcined. $Al_2O_3$-dr* and $Al_2O_3$-calc* are from a different sample batch than used for imaging studies.

## 2.3 Helium pycnometry

He pycnometry was performed with a Pycnomatic ACT EVO (Porotec) at room temperature. At least 10 pulses of He with a gas pressure of 0.2 MPa were applied. The skeletal density ($\rho_{skel}$) was calculated by taking the average value of four consecutive measurements. The experimental error of $\rho_{skel}$ is estimated as 0.002 g cm$^{-3}$. The general characterization methods based porosity information was calculated using eq. 1 with $\rho_{skel}$ from He pycnometry. The total porosity ($\varepsilon_{gen,tot}$) was calculated with the total specific pore volume ($V_{P,tot,Hg}$) from Hg porosimetry. The meso- ($\varepsilon_{gen,m}$) and macroporosity ($\varepsilon_{gen,M}$) were calculated likewise using the specific mesopore ($V_{P,m,Hg}$) and specific macropore volume ($V_{P,M,Hg}$) obtained from Hg porosimetry.

$$\varepsilon_{gen,x} = \frac{V_{P,x,Hg}}{V_{P,tot,Hg} + \frac{1}{\rho_{skel}}} \cdot 100 \quad (1)$$

**Table S 1** Convetional pore characterization results.

| | Ni/$Al_2O_3$ | | Al$_2$O$_3$ | | | |
|---|---|---|---|---|---|---|
| | dried | calcined[a] | dried | calcined | dried* | calcined* |
| $A_{BET}$ / $m^2$ $g^{-1}$ | 5 | 99 | 128 | 91 | - | - |
| $V_{P,tot,N_2}$ / $cm^3$ $g^{-1}$ | 0.02 | 0.31 | 0.22 | 0.24 | - | - |
| $w_{P,N_2}$ / nm | 8.8 | 8.3 | 4.0 | 9.2 | - | - |
| $V_{P,tot,Hg}$ / $cm^3$ $g^{-1}$ | 0.56 | 0.88 | - | 0.67 | 0.80 | 0.71 |
| $V_{P,m,Hg}$ / $cm^3$ $g^{-1}$ | 0.12 | 0.26 | - | 0.26 | 0.13 | 0.21 |
| $V_{P,M,Hg}$ / $cm^3$ $g^{-1}$ | 0.44 | 0.62 | - | 0.41 | 0.67 | 0.50 |
| $w_{P,m,Hg}$ / nm | n.a.[b] | 10.3 | - | 9.4 | 3.7[c] | 9.2 |
| $w_{P,M,Hg}$ / $\mu$m | 1.5 | 1.0 | - | 0.82 | 1.2 | 1.0 |
| $\rho_{skel}$ / g $cm^{-3}$ | 1.944 | 4.028 | - | 3.351 | 1.918 | 3.351 |
| $\varepsilon_{gen,tot}$ / % | 52.1 | 78.0 | - | 55.0 | 60.5 | 70.4 |
| $\varepsilon_{gen,m}$ / % | 11.2 | 23.0 | - | 26.8 | 9.8 | 20.8 |
| $\varepsilon_{gen,M}$ / % | 40.9 | 55.0 | - | 42.3 | 50.7 | 49.6 |

[a] values of the calcined Ni/$Al_2O_3$ sample from ref.[2]
[b] not applicable
[c] pore width smaller than 3.7 nm not detectable
* different sample batch than used for imaging studies

## 2.4 Elemental analysis

Carbon (C), hydrogen (H) and nitrogen (N) elemental analysis (CHN) was performed using a Vario EL MICRO element analyzer (Heraeus). Samples were crimped in aluminum containers and analyzed via heat extraction. The results of the CHN analysis are summarized in Table S 2.

**Table S 2** Results of the CHN-analyis of the dried gel samples and corresponding samples calcined in the laboratory.

| Sample | weighed portion / mg | N / wt.% | C / wt.% | H / wt.% |
|---|---|---|---|---|
| Ni/$Al_2O_3$-dr | 21.021 | 0.00 | 3.40 | 4.76 |
| | 22.077 | 0.00 | 3.44 | 4.73 |
| | 23.003 | 0.00 | 3.40 | 4.67 |
| Ni/$Al_2O_3$-calc | 11.660 | 0.00 | 0.17 | 0.56 |
| | 11.408 | 0.00 | 0.18 | 0.59 |
| | 10.800 | 0.00 | 0.20 | 0.57 |
| $Al_2O_3$-dr | 16.618 | 0.00 | 1.73 | 4.74 |
| | 16.832 | 0.00 | 1.73 | 4.82 |
| | 16.554 | 0.00 | 1.74 | 4.84 |
| $Al_2O_3$-calc | 23.195 | 0.00 | 0.07 | 0.56 |
| | 22.926 | 0.00 | 0.09 | 0.59 |
| | 22.369 | 0.00 | 0.09 | 0.57 |

# 3 *In situ* X-ray ptychography (XRP)

## 3.1 Sample preparation

Wedge-shaped samples for *in situ* X-ray ptychography were prepared from a starting powder particle of the dried gel ($Ni/Al_2O_3$-dr) at a dual beam FIB/SEM instrument[6] via micromanipulation with $Ga^+$ ions. The Ga ion beam current was 5 nA at a voltage of 30 kV. After lift-off of a cuboidal specimen, the sample was mounted on a FIB grid holder and trimmed from all sides to the final wedge-shape to allow for X-ray and TEM analysis on the same sample. For the final surface cleaning and polishing a voltage of 30 kV and a low current of 1 nA was used. After the final preparation the wedge-shaped sample was transferred to a micro-electro-mechanical systems (MEMS) based Wildfire Nano-Chip (DENSsolutions) and glued using ion beam induced deposition (IBID) of a Pt precursor material. Before the sample was attached to the MEMS chip, the SiN membrane on the hole was removed by the ion beam. SEM images were taken at 2 kV at an electron current of 0.1 nA using a secondary electron Everhart-Thornley (ETD) detector in normal and side view with a sample tilt of 0° and 52°, respectively, see, e.g., Figure S 2.

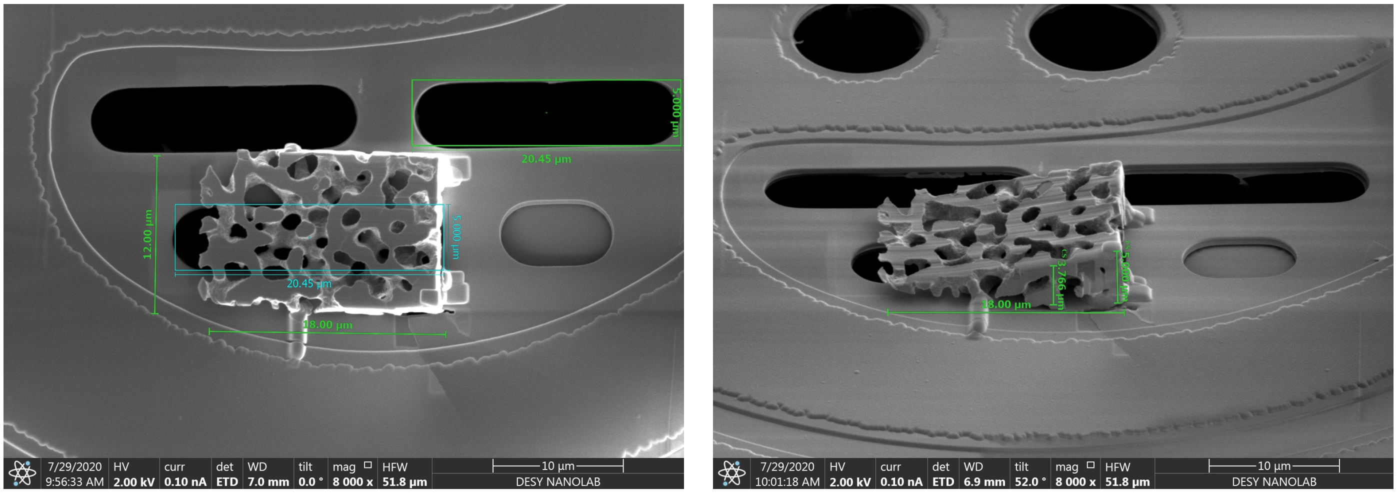


**Figure S 2** SEM images of the wedge-shaped samples used for the *in situ* X-ray ptychography (XRP) experiment. Normal view (left) and tilted view (right) of the FIB-prepared specimen that were prepared from the dried gel ($Ni/Al_2O_3$-dr) are shown to see all relevant dimensions.

## 3.2 Synchrotron experiments

*In situ* 2D X-ray ptychographic imaging (XRP) was performed with the PtyNAMi setup at the Nanoprobe end-station of beamline P06 of the Deutsches Elektronen-Synchrotron DESY (Hamburg, Germany).[7] For *in situ* XRP experiments the nanoreactor setup described by Fam *et al.* was used.[8] The setup consists of the nanoreactor as *in situ* cell shown in Figure S 3. The *in situ* cell contains the MEMS based Wildfire Nano-Chip (DENSsolutions) for electrical heating of the mounted sample. The heating is controlled with a Digiheater temperature controller box (DENSsolutions). The cell further has a gas inlet and outlet, with the outlet connected to an online mass spectrometer (MS), OMNI-Star GSD 320 (Pfeiffer Vacuum). The gas dosing was controlled by a custom made gas-dosing system of mass flow controllers (Bronkhorst). For further details regarding specifications of the *in situ* cell, the reader is referred to ref.[8]

The *in situ* XRP experiments were carried out at an energy of 12 keV. The beam was focused by two sets of 1D focusing diamond lenses in cross geometry, while the sample was scanned out of focus with an effective spot size of $\approx 1.4\,\mu$m on the sample. Far field diffraction patterns were acquired using an EIGER X 4M (Dectris) detector with 2070 x 2167 pixels and a pixelsize of 75 $\mu$m. The sample to detector distance was 3.398 m. Before the *in situ* experiments, the sample was aligned and test scans performed under a constant He flow at room temperature. During *in situ* XRP studies, projections of the sample were obtained for a field of view (FOV) of about 18 x 14 $\mu$m (horizontal x vertical) with a step size of 0.7 $\mu$m and an exposure time of 0.3 s $step^{-1}$. The

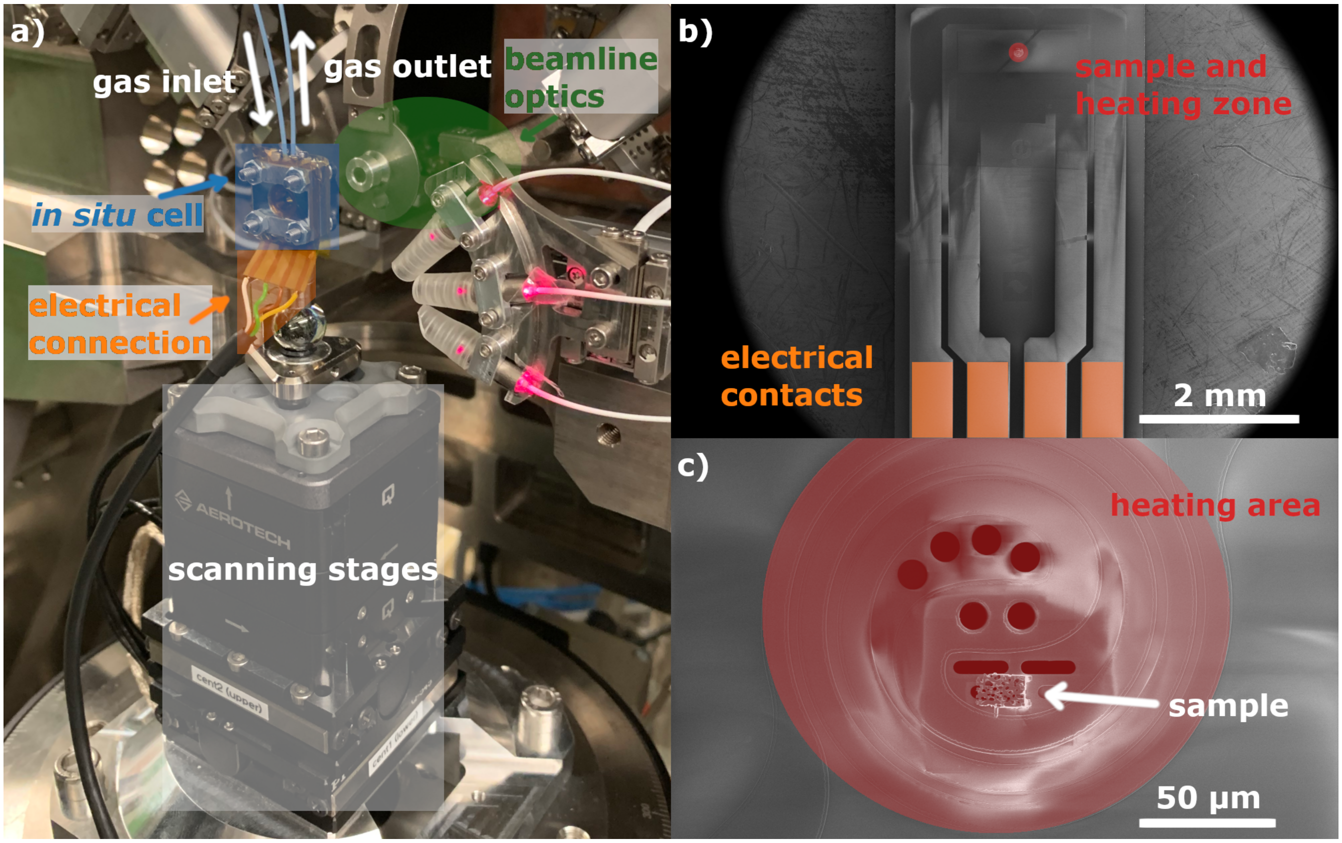


**Figure S 3** Illustration of the samples setup and MEMS chip used for *in situ* X-ray ptychography experiment. (a) nanoreactor *in situ* cell mounted at the beamline as used during the experiments, the outlet is connected to an online mass spectrometer. (b) SEM overview image of the MEMS chip, which is located inside the *in situ* cell of (a). (c) SEM image of the heating zone of the MEMS chip with the sample located on one of the chip windows.

acquisition of one projections required in total about 11 min.
In the *in situ* experiments the $Ni/Al_2O_3$ sample was studied under three different conditions: (i) calcination, (ii) activation and (iii) reaction conditions. A total gas flow of 2 mL min$^{-1}$ was dosed to the *in situ cell*. The gas compositions for each condition are summarized in Table S 3. The *in situ* experiment was carried out as following starting from the dried gel $Ni/Al_2O_3$ sample mounted on the MEMS chip:

- calcination, heating from 303 K to 873 K (30 K steps), holding at 873 K for ≈1 h
- activation, heating from 873 K to 1073 K (50 K steps), holding at 1073 K for ≈1 h, cooling to 673 K
- reaction, at 673 K for ≈1 h, cooling to 303 K (30 K steps )

**Table S 3** Gas composition during the different conditions of the *in situ* XRP experiments of sample $Ni/Al_2O_3$. A total gas flow of 2 mL min$^{-1}$ was used during the *in situ* studies.

| condition | He / mL min$^{-1}$ | $O_2$ / mL min$^{-1}$ | $H_2$ / mL min$^{-1}$ | $CO_2$ / mL min$^{-1}$ |
|---|---|---|---|---|
| calcination | 1.6 | 0.4 | - | - |
| activation | 1.5 | - | 0.5 | - |
| reaction | 1.5 | - | 0.4 | 0.1 |

During the *in situ* experiment the gas composition was constantly analyzed by monitoring the following mass to charge ratios with the assigned compounds in brackets: 2 ($H_2$), 4 (He), 15 ($CH_4$), 16 ($O_2$), 18 ($H_2O$),

28 (CO,$N_2$), 32 ($O_2$), 40 (Ar), 44 ($CO_2$) as shown in Figure S 4. It should be noted that the MS requires a total flow of $\approx 5\,\mathrm{mL\,min^{-1}}$. As the total gas flow during the *in situ* studies was only $2\,\mathrm{mL\,min^{-1}}$, the MS constantly sucked air from the exhaust, which is indicated by the increased Ar and CO/$N_2$ during the *in situ* experiments.

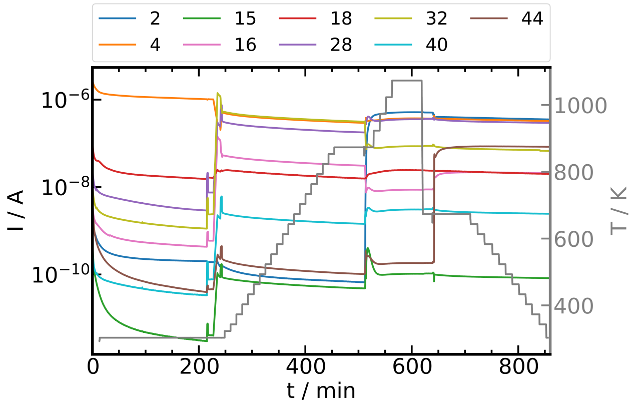


**Figure S 4** Mass spectrometry data as mass to charge ratio $(m/z)$ constantly monitored during the *in situ* XRP experiments of the Ni/$Al_2O_3$ sample together with the MEMS-chip readout temperature.

## 3.3 Ptychographic reconstructions and image analysis

Ptychographic reconstruction of the *in situ* XRP experiments was carried out using an ePIE based algorithm.[9] The diffraction patterns were cropped to 512 x 512 pixels for reconstruction. For each, 1000 iterations of the algorithm were applied. The resulting reconstructed pixel size is 18.6 nm.
The phase contrast projections were further processed using the software ImageJ/Fiji.[10] The phase contrast projections were aligned using the template matching and slice alignment plugin by Tseng *et al.*[11] The open windows of the MEMS-chip above the sample were used as reference for the alignment to avoid any influence of sample changes during the experiment. The width of the sample during the *in situ* experiment was measured manually at a characteristic feature that could be identified in all projections as indicated by the arrows in Figure 2 (main article).
The resolution of the *in situ* XRP experiment was estimated by Fourier ring correlation (FRC)[12] of the last two projections. For FRC the final projections were cropped to 600 x 600 pixels. The results of the FRC analysis are shown in Figure S 5 with an estimated resolution of 67.2 nm using the ½ bit threshold criterion[13]. A movie of the aligned phase contrast projections of the calcination part of the *in situ* XRP experiment is provided as part of the ESI.

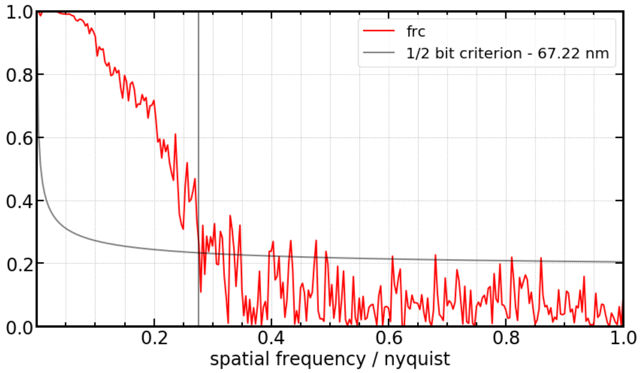


**Figure S 5** FRC analysis of the last two projections for the *in situ* XRP experiment of the Ni/$Al_2O_3$ sample. Reconstructed pixel size 18.6 nm, estimated ½ bit threshold resolution is 67.2 nm for a 600 x 600 pixel cropped area of the sample.

# 4 *Ex situ* ptychographic X-ray computed tomography (PXCT)

## 4.1 Sample preparation

The dried gel samples obtained after synthesis of Ni/$Al_2O_3$-dr and $Al_2O_3$-dr were used as samples for PXCT experiments. The samples were mounted on pure Cu-pins of the OMNY design[14] (without Au coating to avoid Au diffusion at high temperatures) using a dual beam focused-ion beam (FIB, Gallium source), scanning electron microscope (SEM) Strata 400 S FIB/SEM (FEI Company). A suitable particle was attached to a micro-manipulator with Pt-deposition and subsequently transferred and glued to the tip of the Cu-pins by Pt-deposition. FIB preparation time was reduced to a minimum to avoid Pt and Ga contamination of the samples. The resulting mounted particles had a diameter of about 50 $\mu$m and 15 $\mu$m for Ni/$Al_2O_3$-dr and $Al_2O_3$-dr, respectively. SEM images of the mounted samples are shown in Figure S 6.

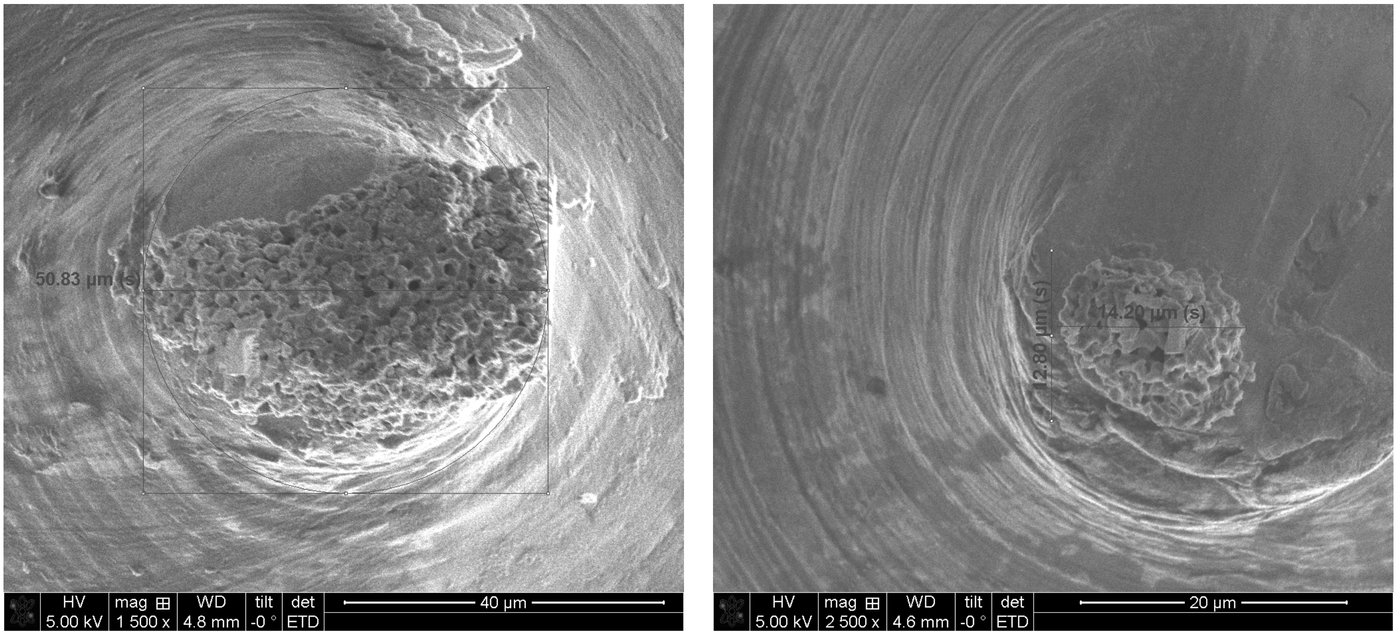


**Figure S 6** Top view SEM images of the dried gel samples mounted on the Cu-pins for PXCT experiments with the Cu-pins below the sample, left: Ni/$Al_2O_3$, right: $Al_2O_3$.

## 4.2 Synchrotron experiments

PXCT experiments were performed at the coherent small angle X-ray scattering (cSAXS) beamline of the Swiss Light Source at the Paul Scherrer Institute (Villigen, Switzerland). The flexible tomography nanoimaging end-station (flOMNI)[15,16] was used for the measurements. The experiments were carried out at room temperature with a constant $N_2$ stream around the sample. PXCT were measured at an energy of 6.2 keV. A Fresnel zone plate (fabricated at the Laboratory for Micro- and Nanotechnology at the Paul Scherrer Institute in Villigen, Switzerland) with a diameter of 200 $\mu$m was used to obtain an illumination size on the sample of about 4 $\mu$m, while the sample was measured 1.5 mm out of the zone plate focus point. Far-field diffraction patterns were acquired with an in-vacuum EIGER 1.5 M detector (Detector group at the Paul Scherrer Institute in Villigen, Switzerland) with 75 $\mu$m pixel size and a sample to detector distance of 5.235 m. For each sample the total tomogram was divided into evenly distributed interlaced sub-tomograms covering a total angular range from 0 to 180 ° in a so-called binary decomposition.[17] Each projection was acquired with the respective field of view (FOV) in $x$ (horizontal) and $y$ (vertical) using a Fermat spiral trajectory[18]. An acquisition time of 25 ms was used for each scanning point. The detailed measurement parameters for each experiment are summarized in Table S 4.

For the experiments, first PXCT of the dried gel samples as shown in Figure S6 were measured. After finishing a tomography scan, the sample was removed from the beamline and calcined mounted on the pin in an external

muffle furnace LE 1/11 (Nabertherm). The sample was heated under flowing air with 10 K $min^{-1}$ to 823 K for 15 min at the highest temperature. A higher heating temperature on the pins is not possible due to reaction of the Cu-pin with air as observed for heating an empty pin to 873 K with 10 K $min^{-1}$ for 2 h. After treatment in the muffle furnace the sample was measured again by PXCT, denoted as the calcined sample.

**Table S 4** Measurement parameters for each PXCT experiment.

| | $Ni/Al_2O_3$ | | $Al_2O_3$ | |
|---|---|---|---|---|
| | dried | calcined | dried | calcined |
| FOV $x$ / $\mu$m | 60 | 48 | 21 | 20 |
| FOV $y$ / $\mu$m | 10 | 10 | 11 | 10 |
| step size / $\mu$m | 1.2 | 1.0 | 1.2 | 1.2 |
| no. sub-tomograms / 1 | 8* | 8 | 16 | 8 |
| no. projections / 1 | 1000 | 1200 | 800 | 500 |
| angular step sub-tomogram / ° | 1.44 | 1.20 | 3.60 | 2.88 |
| estimated dose / $10^7$ Gy | 1.58 | 2.60 | 1.65 | 0.704 |
| total acquisition time / h | 11 | 11.5 | 5 | 2.5 |

* last 6 sub-tomograms used for tomographic reconstruction

## 4.3 Ptychographic and tomographic reconstruction

For ptychographic reconstruction, a region of 700 x 700 pixels (reconstructed pixels size 19.9 nm) and 500 x 500 pixels (reconstructed pixels size 27.9 nm) of the acquired diffraction patterns was used. The obtained reconstructed pixel sizes of the projections were 19.9 nm for $Ni/Al_2O_3$-dr, $Al_2O_3$-calc and $Ni/Al_2O_3$-calc, 27.9 nm for $Al_2O_3$-dr. 300 iterations of the difference map algorithm[19] followed by 300 iterations of maximum likelihood refinement[20,21] were used to reconstruct the projections. The obtained 2D projection series of the complex-valued transmission of the sample was first aligned according to Guizar-Sicairos *et al.* and Odstrčil *et al.*[22,23] The series was aligned in the following steps: (i) cross-correlation, (ii) mass fluctuation based vertical alignment and (iii) tomographic consistency alignment[24]. The aligned phase projection series was subsequently tomographically reconstructed with a filtered-back projection algorithm employing a Ram-Lak filter. The resulting tomogram with the refractive index decrement ($\delta(r)$) contrast was used for further analysis. The electron density ($N_e(r)$) tomogram can be calculated from the $\delta(r)$ tomogram as described in eq. 11 and section 4.6. 3D Fourier shell correlation (FSC) was used to estimate the resolution of the obtained tomograms.[13] The tomogram dataset was divided in two independent subtomograms with double angular spacing, which were reconstructed individually. On these two independent reconstructed tomograms FSC was performed to estimate the effective resolution using the ½ bit threshold criterion.[13] The FSC analysis for the $Ni/Al_2O_3$ and $Al_2O_3$ samples are shown in Figures S 7,8, respectively.
Potential X-ray beam induced sample changes of the dried gels was checked during the tomographic reconstructions. For $Ni/Al_2O_3$-dr slight sample changes were observed for the first two subtomograms. This is also indicated by comparing the resolution from FSC analysis of the reconstructed tomograms using 6 (91.2 nm, Figures S 7) or 8 (102.7 nm) sub-tomograms. Therefore the first two subtomograms of $Ni/Al_2O_3$-dr were excluded to reconstruct the final tomogram. In case of $Al_2O_3$-dr the FSC analysis of tomographic reconstructions of the first 8 (51.6 nm) and the full 16 (49.6 nm, Figures S 8) sub-tomograms revealed not such pronounced changes. Therefore, all 16 sub-tomograms were used to obtain the final tomographic reconstructions. For the calcined samples no X-ray beam induced changes could be identified, which indicates that the samples changes could be related to the organic and solvent residues.

The reconstructed $\delta(r)$ contrast tomograms are available under:
DOI: 10.5445/IR/1000140344

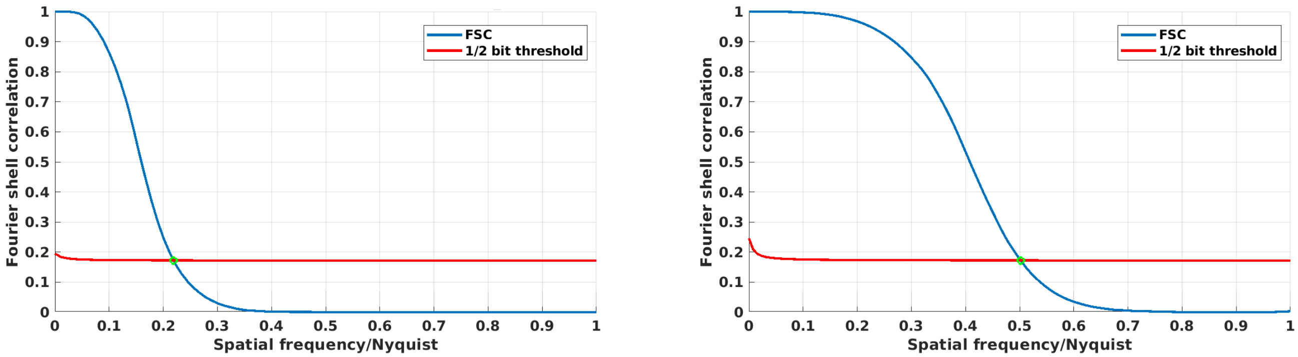


**Figure S 7** FSC analysis of the PXCT of the Ni/$Al_2O_3$ samples. Left: Ni/$Al_2O_3$-dr reconstructed voxel size 19.9 nm, estimated resolution 91.2 nm, right: Ni/$Al_2O_3$-calc reconstructed voxel size 27.9 nm, estimated resolution 55.6 nm.

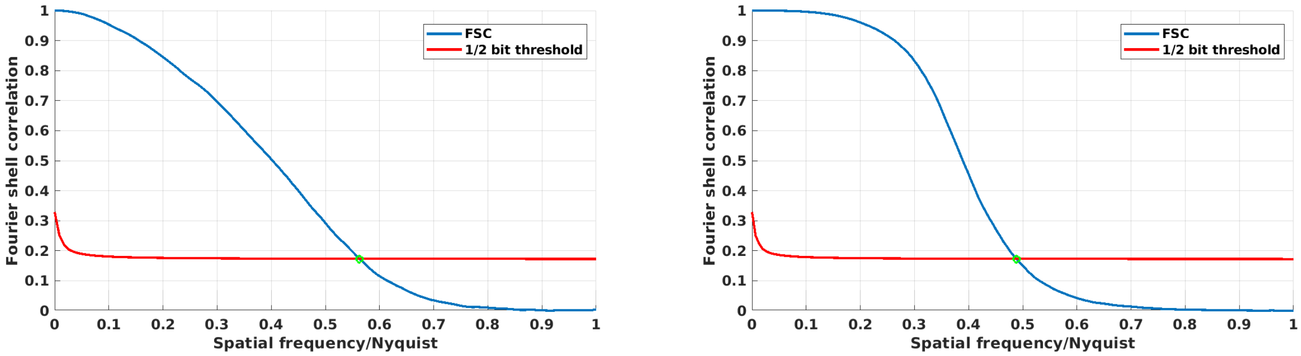


**Figure S 8** FSC analysis of the PXCT of the $Al_2O_3$ samples. Left: $Al_2O_3$-dr reconstructed voxel size 27.9 nm, estimated resolution 49.6 nm, right: $Al_2O_3$-calc reconstructed voxel size 19.9 nm, estimated resolution 40.9 nm.

## 4.4 Image analysis

Image analysis of the PXCT data was performed using Avizo 9.7.0 (FEI SAS, Thermo Fisher Scientific). For the analysis of the $N_e$ tomograms, the whole sample particle volume was masked from the surrounding air to obtain the *particle* label by using the *Lasso* and *Interpolation* tools within the segmentation editor of the software. The *particle* label was then further segmented by thresholding into *pore*, *material* and *contamination* labels, see main article Figure 3. The *pore* label was separated into individual pores by the *Separate Objects* function to obtain the *separated pores* label. An *Opening* module was applied on the *separated pores* to remove single voxel artefacts from the separation. The resulting *separated pores* label is shown in Figure 4, main article. For quantitative analysis of the tomograms the *Label Analysis* module was applied on the *pore*, *separated pores* and *material* labels of the tomograms. The *pore* and *material* labels are used to calculate the macroporosity of the samples, while the *separated pores* label is used for analysis of the equivalent pore diameter.
The *pore* label was further analyzed by applying an *Auto Skeleton* module to retrieve a pore network model of the macropores within the particle. Quantitative information about the pore network model were obtained by applying the *Spatial Graph Statistics* module. The resulting spatial graph of the pore network model contains several sub-graphs. The sub-graphs consist of nodes, which are connected by segments. For all samples in this study one main spatial graph was obtained and the detailed analysis is limited to these main graphs. Other detected graphs consisted only of few pore nodes and segments located at the surface of the particle volume, which were assigned to surface artefacts. The presence of one main graph additionally confirms the presence of a well connected macropore structure in all samples. The statistical analysis of the main spatial graphs provides information about the coordinates of each node and its coordination number (*CN*). Furthermore, for each

segment tortuosity ($\tau$), cylindrical radius ($r_{pore}$) / diameter ($d_{pore}$), volume ($V_{pore}$) and orientation information can be retrieved.

## 4.5 Pore system characterization

**Porosity calculations**

Information about the macropores of the samples can be directly retrieved from quantitative image analysis of the PXCT. The macroporosity ($\varepsilon_M$) can be calculated from the total pore $V_{M,P}$ and material $V_{mlabel}$ labels volumes as following:

$$\varepsilon_M = \frac{V_{M,P}}{V_{M,P} + V_{mlabel}} \tag{2}$$

while $V_{M,P}$ is also corresponding to the macropore volume of the sample. The total porosity ($\varepsilon_{tot}$) of the particle is defined as following:

$$\varepsilon_{tot} = \varepsilon_M + \varepsilon_m \tag{3}$$

In general the mesoporosity ($\varepsilon_m$) can be defined with the mesopore volume ($V_{m,P}$), macropore volume ($V_{M,P}$) and solid volume ($V_S$) as:

$$\varepsilon_m = \frac{V_{m,P}}{V_{m,P} + V_{M,P} + V_S} \tag{4}$$

while $V_{m,P}$ and $V_S$ cannot be directly resolved by PXCT in the experiments The sum of the volumes corresponds to the material label volume ($V_{mlabel}$):

$$V_{mlabel} = V_{m,P} + V_S \tag{5}$$

Thus, $\varepsilon_m$ cannot be directly retrieved from the label based image analysis. However, $\varepsilon_m$ can be calculated from the analysis of the electron density of the material label of the samples. The material label porosity ($\varepsilon_{mat}$), which can be obtained from the electron density analysis in eq. 14 can be also defined based on the volumes:

$$\varepsilon_{mat} = \frac{V_{m,P}}{V_{mlabel}} \tag{6}$$

Thus $V_{m,P}$ can be expressed as:

$$V_{m,P} = \varepsilon_{mat} \cdot V_{mlabel} \tag{7}$$

Furthermore, the macropore volume $V_{M,P}$ can be described by the known $\varepsilon_M$ from eq. 2 as:

$$V_{M,P} = \frac{\varepsilon_M \cdot V_{mlabel}}{(1 - \varepsilon_M)} \tag{8}$$

With eq. 7 and 8 we can substitute the volumes in eq. 4 to retrieve an expression for $\varepsilon_m$ based on $\varepsilon_M$ and $\varepsilon_{mat}$:

$$\varepsilon_m = \frac{\varepsilon_{mat}}{1 + \left(\frac{\varepsilon_M}{1 - \varepsilon_M}\right)} \tag{9}$$

**Pore radius/diameter analysis**

The diameter of the pores was analyzed via two different ways. Based on the quantitative label analysis an equivalent pore diameter ($d_{eq}$) can be defined based on the individual pore volume ($V_{ind,P}$) as:

$$d_{eq} = \sqrt[3]{\frac{6V_{ind,P}}{\pi}} \tag{10}$$

The $d_{eq}$ probes the total volume of the separated pores and can be seen as a measure of the pore body. On the other hand the pore network model provides information about the cylindrical $r_{pore}$ of each segment. $r_{pore}$ obtained from the spatial graph analysis rather probe the pore throat than the pore body.

**Further information from the pore network model**

The pore network model obtained from the spatial graph analysis in Avizo provides further measures about the pore system that can be relevant in respect of mass transport properties. For an analysis of the location of nodes and pores (segments) within the particle, the center is defined as the gravity center of the whole *particle* label. With the coordinates of the gravity center we can calculate the distance to center ($d_{center}$) for each node and segment. As a segment connects two nodes, the coordinates of the segment is approximated as the middle point of the vector directly connecting the nodes. Average measures depending on $d_{center}$ were computed for binned spherical shells of 200 nm and 100 nm thickness for $Ni/Al_2O_3$ and $Al_2O_3$, respectively. The average coordination number ($\overline{CN}$) of the nodes depending on $d_{center}$ are shown in Figure S 9.

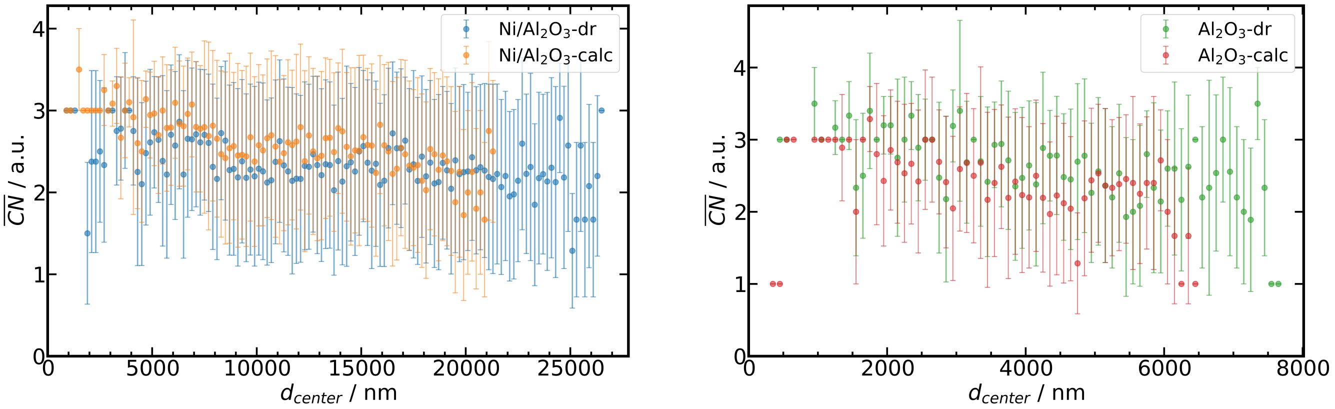


**Figure S 9** Average coordination number ($\overline{CN}$) of the nodes with standard deviation ($\sigma$) as errorbars depending on the distance to the particle center ($d_{center}$), left: $Ni/Al_2O_3$ ($d_{center}$ binning 200 nm), right: $Al_2O_3$ ($d_{center}$ binning 100 nm).

Besides $\overline{CN}$, the average tortuosity ($\overline{\tau}$) of the segments was analyzed depending on $d_{center}$ as shown in Figure S 10. Additionally, the distribution of the tortuosity $\tau$ weighted by the volume of the respective segment is shown in Figure S 11. It should be noted that the discussed $\tau$ represents single segments and not a whole path through the particle. Therefore we refer this $\tau$ as a microscopic tortuosity. However, with more detailed pore network analysis whole paths through the sample can be analyzed to retrieve a macroscopic tortuosity. For both systems $Ni/Al_2O_3$ and $Al_2O_3$ no significant differences of $\overline{CN}$ and $\overline{\tau}$ between the dried gel and calcined samples can be identified. Also the analysis of $\overline{CN}$ and $\overline{\tau}$ in respect to $d_{center}$ shows no significant trends in regions that are statistically trustful. It should be noted that the for statistical analysis artefacts might arise very close to the particle center and particle surface. This can be clearly observed in Figure S 9 for $\overline{CN}$ close to the center. These are mainly caused by undersampling, which can also be identified by missing error bars pointing out single values in the respective analyzed shell.

Additionally to the analysis of $\overline{CN}$ and $\overline{\tau}$, the distribution of $r_{pore}$ was analyzed depending on $d_{center}$. A 2D histogram of the relative normalized volume ($V_{rel,norm}$) weighted $r_{pore}$ distribution was computed. Furthermore, a kernel density estimate (KDE) was computed of the 2D histogram as implemented in the seaborn python library.[25] With the KDE a contour was calculated enclosing 75 % of the volume weighted pores. The 2D histograms together with the 75 % contours are shown in Figure S 12,13 for $Ni/Al_2O_3$ and$Al_2O_3$, respectively.

To show the potential and amount of information available in PXCT studies of pore structures, we further performed a correlation analysis of the normalized pore volume ($V_{rel,norm}$), $r_{pore}$, $d_{center}$ and $\tau$. The results of the correlation analysis is shown in Figure S 14,15 for $Ni/Al_2O_3$ and $Al_2O_3$, respectively. The scatter plots of the correlation analysis contain 4D information (2 axes, scatter size and scatter color) and can be used to identify potential clusters with certain properties within the studied particles. Exemplary, for $\tau$ (scatter color) no obvious clusters can be observed, as already indicated by the analysis of $\overline{\tau}$ depending on $d_{center}$ shown in Figure S 10. The correlation analysis might unravel correlation, which might not be easily identified in common x-y data representation.

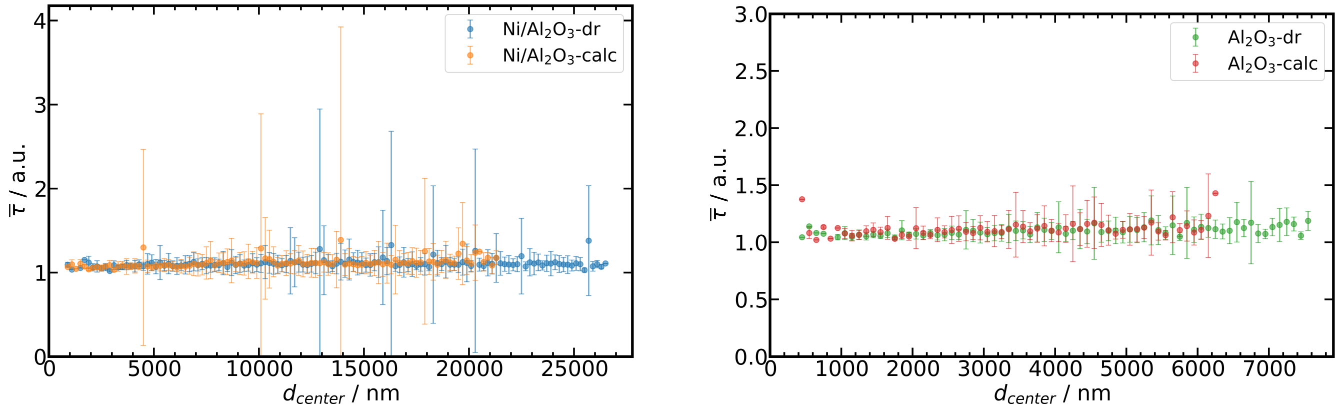


**Figure S 10** Average tortuosity ($\overline{\tau}$) of the segments with standard deviation ($\sigma$) as errorbars depending on the distance to the particle center ($d_{center}$), left: Ni/$Al_2O_3$ ($d_{center}$ binning 200 nm), right: $Al_2O_3$ ($d_{center}$ binning 100 nm).

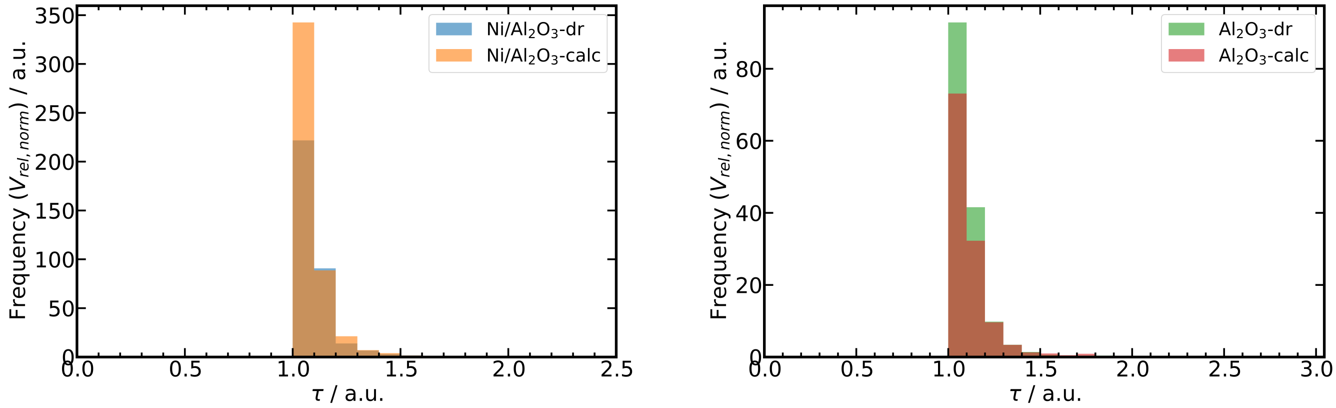


**Figure S 11** Segment tortuosity ($\tau$) distribution weighted by the normalized segment volume ($V_{rel,norm}$), left: Ni/$Al_2O_3$, right: $Al_2O_3$.

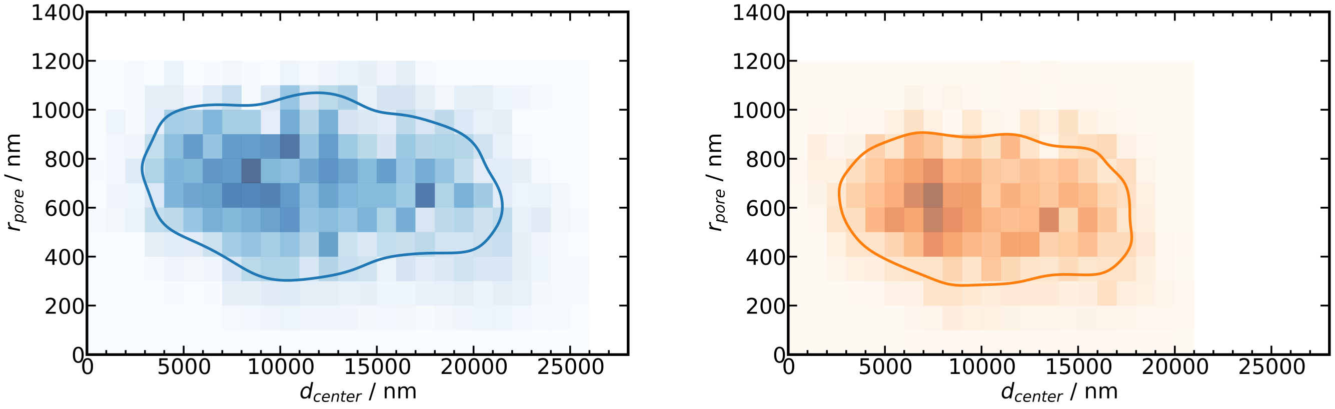


**Figure S 12** 2D histogram of the volume ($V_{rel,norm}$) weighted segment based pore radius $r_{pore}$ distribution for binning of $r_{pore} = 100$ nm and $d_{center} = 1000$ nm together with the KDE based 75 % contour, left: Ni/$Al_2O_3$-dr, right: Ni/$Al_2O_3$-calc.

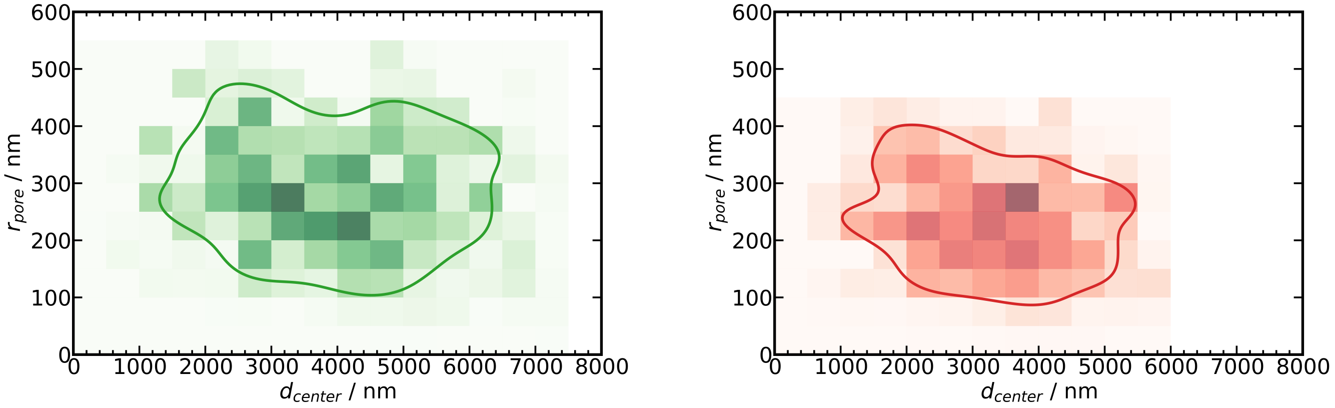


**Figure S 13** 2D histogram of the volume ($V_{rel,norm}$) weighted segment based pore radius $r_{pore}$ distribution for binning of $r_{pore} = 50$ nm and $d_{center} = 500$ nm together with the KDE based 75 % contour, left: $Al_2O_3$-dr, right: $Al_2O_3$-calc.

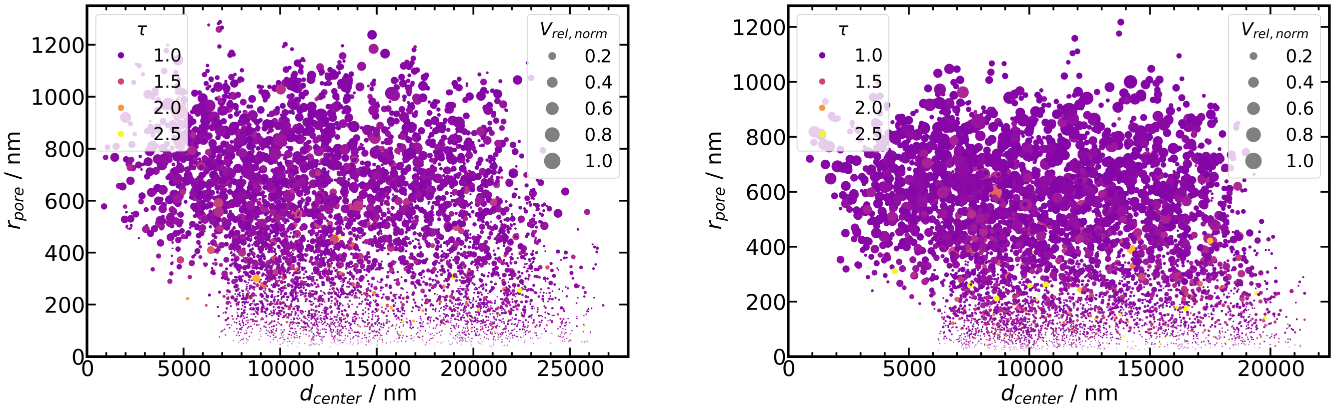


**Figure S 14** Scatter plot for correlation analysis of the segments radius ($r_{pore}$), distance to particle center ($d_{center}$) with the normalized segment volume ($V_{rel,norm}$, scatter size) and segment tortuosity ($\tau$, scatter color), left: Ni/$Al_2O_3$-dr, right: Ni/$Al_2O_3$-calc.

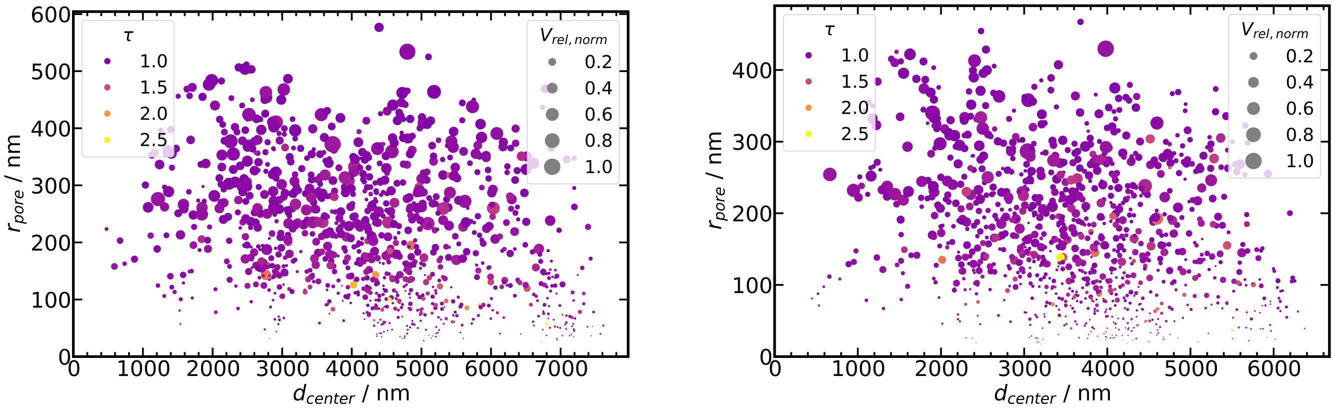


**Figure S 15** Scatter plot for correlation analysis of the segments radius ($r_{pore}$), distance to particle center ($d_{center}$) with the normalized segment volume ($V_{rel,norm}$, scatter size) and segment tortuosity ($\tau$, scatter color), left: $Al_2O_3$-dr, right: $Al_2O_3$-calc.

## 4.6 Electron density analysis

The electron density ($N_e$) of the PXCT was obtained from the phase contrast tomograms as described in ref.[26,27] and in the following equation:

$$N_e(r) = \frac{2\pi \cdot \delta(r)}{\lambda^2 \cdot r_0} \tag{11}$$

with $\lambda$ as the wavelength and $r_0$ as the classical electron radius. From the $N_e$ tomograms the mass density $\rho$ tomograms can be calculated as adapted from ref.[27]:

$$\rho(r) = \frac{N_e(r) \cdot M}{N_A \cdot Z_{UC}} \tag{12}$$

$$\rho = N_e \cdot f_{mass} \tag{13}$$

with $M$ as the molar mass of the unit cell, $N_A$ as Avogadro's constant and $Z_{UC}$ as the number of electrons per unit cell, which are defined as the mass density factor ($f_{mass}$). The $N_e$ of the material label with $f_{mass}$ can be used to calculate the mesoporosity $\varepsilon_m$ of the sample particle. First the porosity of only the material label can be calculated as following:

$$\varepsilon_{mat} = 1 - \frac{N_{e,mode} \cdot f_{mass}}{\rho_{theo}} \tag{14}$$

The mode of the $N_e$ distribution of the whole material label is used to approximate the average material porosity $\varepsilon_{mat}$, while knowledge about the theoretical mass density $\rho_{theo}$ is required. The information about $\rho_{theo}$ can be either retrieved as the X-ray density of the unit cell for the case a crystal structure of the material is known or approximated by experimental measurement of the skeletal density using He pycnometry. The $N_e$ based $\varepsilon_{mat}$ can be readily used to calculate $\varepsilon_m$ if $\varepsilon_M$ is known from quantitative label analysis.
The $N_e$ mode was determined from the normalized $N_e$ distribution of the material labels as shown in Figure S 16 and summarized in Table S 5. From the mode the respective $\varepsilon_{mat}$ was calculated according to eq. 14, with $f_{mass}$ and $\rho_{theo}$ as summarized in Table S 5. For the dried and calcined states the same chemical formula were assumed for each system as $Ni_{0.43}Al_2O_4$ and $Al_2O_3$, respectively. $f_{mass}$ were calculated according to eq. 12, assuming a defect spinel structures for both systems. $\rho_{theo}$ for the Ni containing sample was determined by He pycnometry, as no exact crystal structure for the sample composition is known. For $Al_2O_3$, $\rho_{theo}$ is the X-ray density based on the crystal structure of $\gamma$-$Al_2O_3$ as retrieved from ref.[28]

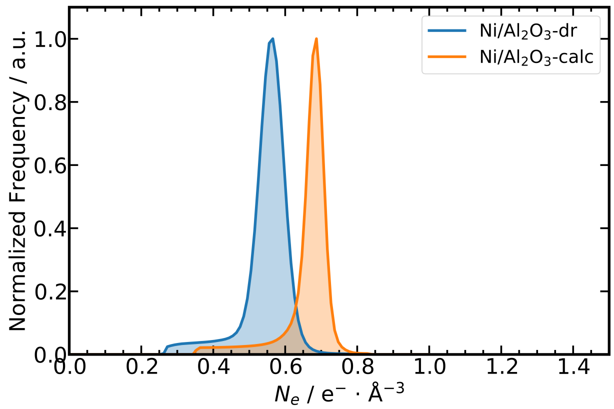


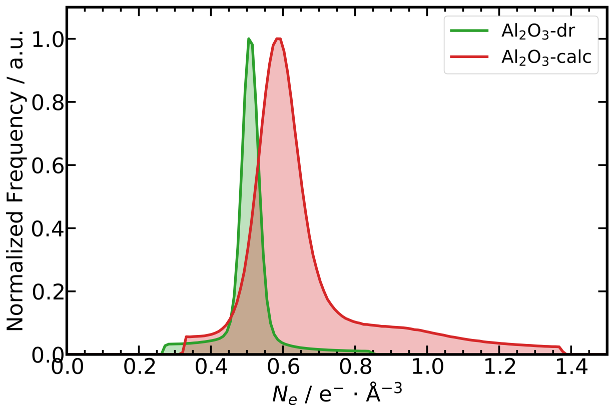


**Figure S 16** Electron density ($N_e$) distribution for the *material* label of the dried and calcined samples, left: Ni/$Al_2O_3$, right: $Al_2O_3$.

**Table S 5** Summarized results of the analysis of the electron density ($N_e$) distribution of the *material* label of the dried and calcined samples of Ni/$Al_2O_3$ and $Al_2O_3$. Material label porosity ($\varepsilon_{mat}$); mass factor ($f_{mass}$) and theoretical mass density ($\rho_{theo}$) used to calculate $\varepsilon_{mat}$; mesoporosity ($\varepsilon_m$) calculated from $\varepsilon_{mat}$, macroporosity ($\varepsilon_M$) from image analysis and total porosity ($\varepsilon_{tot}$) obtained from image and $N_e$ analysis.

| | Ni/$Al_2O_3$ | | $Al_2O_3$ | |
|---|---|---|---|---|
| | dried | calcined | dried | calcined |
| mode $N_e$ / $e^-$ Å$^{-3}$ | 0.561 | 0..683 | 0.511 | 0.591 |
| $\varepsilon_{mat}$ / % | 52.5 | 42.2 | 52.7 | 45.3 |
| $f_{mass}$ / $10^{23}$ g | 3.407 | 3.407 | 3.386 | 3.386 |
| $\rho_{theo}$ / g cm$^{-3}$ | 4.028 | 4.028 | 3.66 | 3.66 |
| $\varepsilon_m$ / % | 29.4 | 23.4 | 34.3 | 30.1 |
| $\varepsilon_M$ / % | 43.9 | 44.5 | 34.9 | 33.6 |
| $\varepsilon_{tot}$ / % | 73.3 | 67.9 | 69.2 | 63.7 |

# 5 Statistical Analysis

Details regarding the data (pre-)processing are reported for each of the techniques in the supplementary information.

In general, image analysis was performed using the software Avizo 9.7.0 (FEI SAS, Thermo Fisher Scientific). The obtained data from image analysis (Table S 6) were further processed and visualized using NumPy[29], matplotlib[30] and seaborn[25] libraries. Each pore contained information about its volume, equivalent spherical diameter $d_{eq}$ and location in $x, y, z$ based on the gravimetric center of the pore. Each node contained information about the location in $x, y, z$ and coordination number $CN$. Every segment contained information about the cylindrical segment radius/diameter $r_{pore}/d_{pore}$, segment volume, tortuosity $\tau$, segment length and the ID of the connected nodes.

Analyzed data of the image analysis are presented as relative volume weighted distributions, where applicable as mean values with standard deviation as error bars or as stated otherwise. For scatter plots in Figure 6 and Figure S 12,13 a kernel density estimation (KDE) as implemented in seaborn[25] was performed and the KDE-based contour enclosing 75 % of the volume weighted pores is highlighted. Were applicable, values are reported as mean values with standard deviation.

The mean and standard deviation for the slice based electron density analysis were directly obtained from image analysis in Avizo 9.7.0.

**Table S 6** Number of data points derived from the image analysis of the PXCT using the software Avizo for the two different pore network models.

| | $Ni/Al_2O_3$ | | $Al_2O_3$ | |
|---|---|---|---|---|
| | dried | calcined | dried | calcined |
| | Separate individually labeled pores model | | | |
| number of pores / [1] | 971 | 880 | 217 | 265 |
| | Spatial graph pore network model | | | |
| number of segments / [1] | 6415 | 5654 | 1078 | 1119 |
| number of nodes / [1] | 5553 | 4409 | 823 | 935 |

## References


[1] J. Herwig, J. Titus, J. Kullmann, N. Wilde, T. Hahn, R. Gläser, D. Enke, *ACS Omega* **2018**, *3*, 1 1201.

[2] S. Weber, K. L. Abel, R. T. Zimmermann, X. Huang, J. Bremer, L. K. Rihko-Struckmann, D. Batey, S. Cipiccia, J. Titus, D. Poppitz, C. Kübel, K. Sundmacher, R. Gläser, T. L. Sheppard, *Catalysts* **2020**, *10*, 12 1.

[3] S. Brunauer, P. H. Emmett, E. Teller, *J. Am. Chem. Soc.* **1938**, *60*, 2 309.

[4] E. P. Barrett, L. G. Joyner, P. P. Halenda, *J. Am. Chem. Soc.* **1951**, *73*, 2 373.

[5] E. W. Washburn, *Phys. Rev.* **1921**, *17*, 3 273.

[6] A. Stierle, T. F. Keller, H. Noei, V. Vonk, R. Roehlsberger, *JLSRF* **2016**, *2* A76.

[7] A. Schropp, R. Dohrmann, S. Botta, D. Bruckner, M. Kahnt, M. Lyubomirskiy, C. Ossig, M. Scholz, M. Seyrich, M. E. Stuckelberger, P. Wiljes, F. Wittwer, J. Garrevoet, G. Falkenberg, Y. Fam, T. L. Sheppard, J.-D. Grunwaldt, C. G. Schroer, *J. Appl. Cryst.* **2020**, *53* 957.

[8] Y. Fam, T. L. Sheppard, J. Becher, D. Scherhaufer, H. Lambach, S. Kulkarni, T. F. Keller, A. Wittstock, F. Wittwer, M. Seyrich, D. Brueckner, M. Kahnt, X. Yang, A. Schropp, A. Stierle, C. G. Schroer, J.-D. Grunwaldt, *J. Synchrotron Rad.* **2019**, *26* 1769.

[9] A. M. Maiden, J. M. Rodenburg, *Ultramicroscopy* **2009**, *109*, 10 1256.

[10] J. Schindelin, I. Arganda-Carreras, E. Frise, V. Kaynig, M. Longair, T. Pietzsch, S. Preibisch, C. Rueden, S. Saalfeld, B. Schmid, J.-Y. Tinevez, D. J. White, V. Hartenstein, K. Eliceiri, P. Tomancak, A. Cardona, *Nat. Methods* **2012**, *9* 676.

[11] Q. Tseng, E. Duchemin-Pelletier, A. Deshiere, M. Balland, H. Guilloud, O. Filhol, M. Theŕy, *PNAS* **2012**, *109*, 5 1506.

[12] N. Banterle, K. H. Bui, E. A. Lemke, M. Beck, *J. Struct. Biol.* **2013**, *183*, 3 363.

[13] M. van Heel, M. Schatz, *J. Struct. Biol.* **2005**, *151*, 3 250.

[14] M. Holler, J. Raabe, R. Wepf, S. H. Shahmoradian, A. Diaz, B. Sarafimov, T. Lachat, H. Walther, M. Vitins, *Rev. Sci. Instrum.* **2017**, *88*, 11 113701.

[15] M. Holler, J. Raabe, A. Diaz, M. Guizar-Sicairos, C. Quitmann, A. Menzel, O. Bunk, *Rev. Sci. Instrum.* **2012**, *83*, 7 073703.

[16] M. Holler, A. Diaz, M. Guizar-Sicairos, P. Karvinen, E. Färm, E. Härkönen, M. Ritala, A. Menzel, J. Raabe, O. Bunk, *Sci. Rep.* **2014**, *4* 1.

[17] A. Kaestner, B. Münch, P. Trtik, L. Butler, *Opt. Eng.* **2011**, *50*, 12 123201.

[18] X. Huang, H. Yan, R. Harder, Y. Hwu, I. K. Robinson, Y. S. Chu, *Optics Express* **2014**, *22*, 10 12634.

[19] P. Thibault, M. Dierolf, A. Menzel, O. Bunk, C. David, F. Pfeiffer, *Science* **2008**, *321*, July 379.

[20] M. Guizar-Sicairos, J. R. Fienup, *Opt. Express* **2008**, *16*, 10 7264.

[21] P. Thibault, M. Guizar-Sicairos, *New J.Phys.* **2012**, *14* 063004.

[22] M. Guizar-Sicairos, A. Diaz, M. Holler, M. S. Lucas, A. Menzel, R. A. Wepf, O. Bunk, *Opt. Express* **2011**, *19*, 22 21345.

[23] M. Odstrčil, M. Holler, J. Raabe, M. Guizar-Sicairos, *Opt. Express* **2019**, *27*, 25 36637.

[24] M. Guizar-Sicairos, J. J. Boon, K. Mader, A. Diaz, A. Menzel, O. Bunk, *Optica* **2015**, *2*, 3 259.

[25] M. L. Waskom, *J. Open Source Softw.* **2021**, *6*, 60 3021.

[26] M. Dierolf, A. Menzel, P. Thibault, P. Schneider, C. M. Kewish, R. Wepf, O. Bunk, F. Pfeiffer, *Nature* **2010**, *467*, 7314 436.

[27] A. Diaz, P. Trtik, M. Guizar-Sicairos, A. Menzel, P. Thibault, O. Bunk, *Phys. Rev. B Condens. Matter* **2012**, *85*, 2 1.

[28] R.-S. Zhou, R. L. Snyder, *Acta Cryst. B* **1991**, *47*, 5 617.

[29] C. R. Harris, K. J. Millman, S. J. van der Walt, R. Gommers, P. Virtanen, D. Cournapeau, E. Wieser, J. Taylor, S. Berg, N. J. Smith, R. Kern, M. Picus, S. Hoyer, M. H. van Kerkwijk, M. Brett, A. Haldane, J. F. del Río, M. Wiebe, P. Peterson, P. Gérard-Marchant, K. Sheppard, T. Reddy, W. Weckesser, H. Abbasi, C. Gohlke, T. E. Oliphant, *Nature* **2020**, *585*, 7825 357.

[30] J. D. Hunter, *Comput. Sci. Eng.* **2007**, *9*, 3 90.
